# Silica nanospheres KCC-1 as a good catalyst for the preparation of 2-amino-4H-chromenes by ultrasonic irradiation

**DOI:** 10.1038/s41598-022-05993-3

**Published:** 2022-02-11

**Authors:** Hourieh Sadat Oboudatian, Javad Safaei-Ghomi

**Affiliations:** Department of Organic Chemistry, Faculty of Chemistry, University of Kashan, Kashan, Islamic Republic of Iran

**Keywords:** Chemistry, Catalysis, Green chemistry, Organic chemistry

## Abstract

Fibrous nano-silica sphere (KCC-1) has appeared as a good and efficient catalyst for ultrasonic irradiation conditions in chemical reactions. This catalyst has the unique properties such as a fibrous surface morphology, high surface area and high mechanical stability. The results indicated that the KCC-1 nanocatalyst could be used as high-performance catalysts under high temperature and pressure condition in organic reaction under ultrasonic irradiation. Morphology, structure, and composition of the fibrous nano-silica sphere were described by N2 adsorption–desorption analysis, scanning electron microscopy (SEM), transmission electron microscopy (TEM), X-ray powder diffraction (XRD), thermogravimetric analysis (TGA) and Fourier-transform infrared spectroscopy (FT-IR). In this work, we used KCC-1@NH_2_ nanosilica as a basic catalyst for the preparation of chromenes under ultrasonic irradiation conditions for the first time. The recyclability, nontoxicity and high stability of the catalyst, combined with low reaction times and excellent yields, make the present protocol very useful for the synthesis of the title products under ultrasonic conditions. The produced products were confirmed via ^1^H NMR, ^13^C NMR, FT-IR analysis.

## Introduction

**Figure 1:**
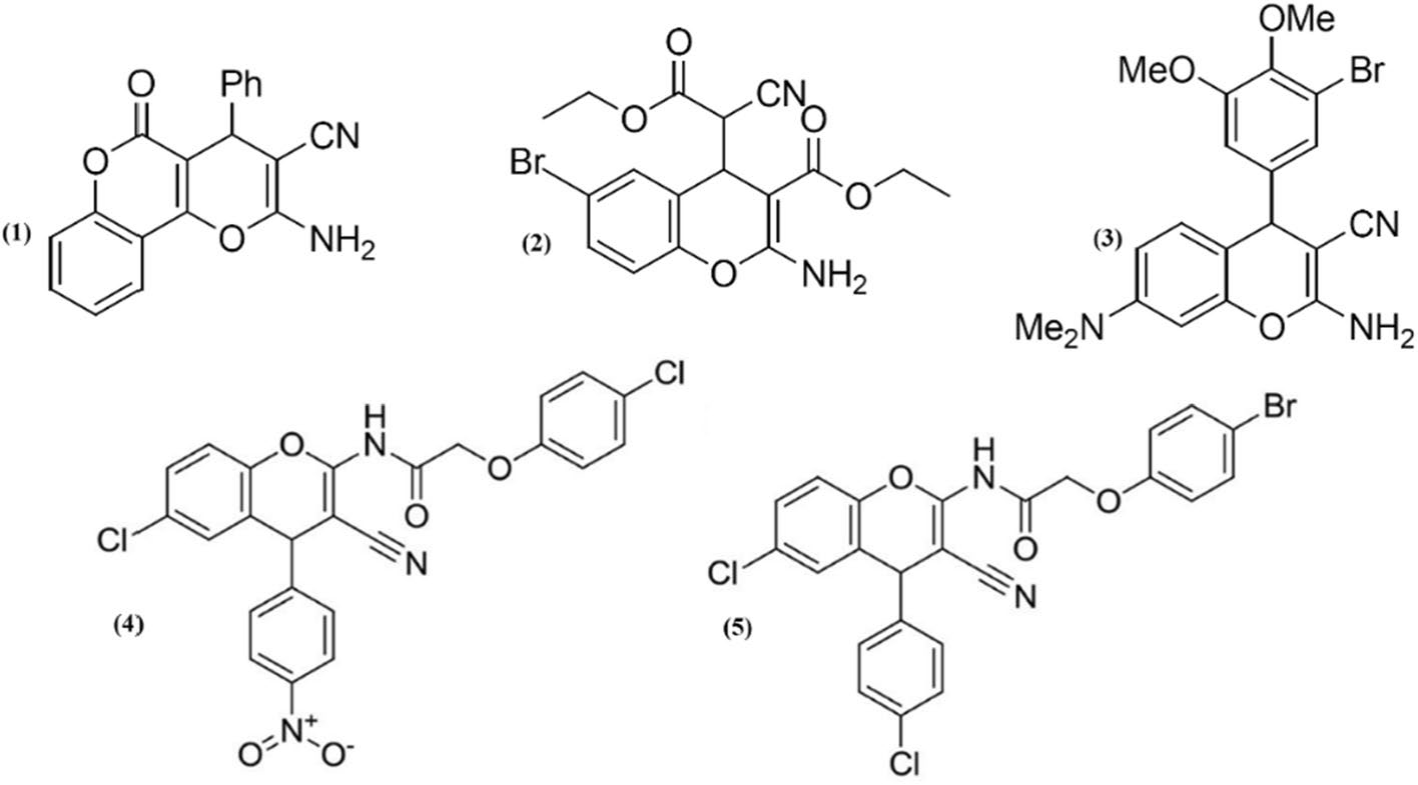
Structures of some 2-amino-4H-chromenes with diverse biological activities.

In recent years, many studies have been concentrated on increasing the performance of organic and heterogeneous catalytic synthesis because of their applicable importance in synthesis of medicinal compounds by green methods. One of the progressive strategies which have recently attracted considerable attention is the usage of ultrasound conditions with heterogeneous catalysts. 2-Amino-4H-chromenes are an important class for further development in medicinal and organic synthesis studies due to their potency and a wide spectrum of biological activities including cancer therapy ^1,2^, antiviral ^3,4^, antitumor ^5^ and sex hormone ^6^. For example (Fig. 1), pyranopyranone (1) as an ancestor for the blood anticoagulant warfarin ^7^, (4H-chromen-4-yl)cyanoacetate (2) as inhibitor of Bcl-2 protein and apoptosis inducer ^8^ and benzopyrane (3) has been known for anticancer therapeutic ^9^. Also compounds 4 and 5 showed in Fig. 1 the maximum inhibitory effect against the HT29 human colon cancer cells ^10^. Chromenes have been used for the treatment of different diseases of connective tissues, diabetes, psoriasis, pernicious anemia, ulcerous colitis, and chronic hepatitis ^11^. These derivatives are employed as a building block of many natural products ^12,13^, food additives, Pigments, Pesticides, cosmetic agents and potentially biodegradable agrochemicals ^14^.

The preparation of 2-Amino-4H-Chromenes has been reported using various conditions and catalysts such as piperidine ^15,16^, piperazine ^17^, triethyl amine ^18^, IL ^19^, MCM-41 ^20^, K_2_CO_3_^21^. Most of the reported methods need long reaction times, use of toxic solvents, low yields, non-reusable catalysts and stoichiometric reagents. In the present paper, we afford to introduce a new efficient method for the preparation of these medicinal compounds by utilizing acoustic cavitation. The synthesis of substances under ultrasonic condition not only requires fewer catalysts and solvents, but also meets the environmental requirements more powerfully. In Continuing our previous researches ^22^, to investigate the performance of ultrasonic irradiation, we report the fabrication of Co3O4@PPIL-Mo as a catalyst and for the synthesis of mono-spiro derivatives under ultrasonic irradiation. Also, literatures have been reported for the synthesis of spiroindolines ^23^, pyridopyrimidines ^24^, 2, 3-dihydroquinazolin-4 (1H)-ones ^25^ and oxidation of benzyl alcohol under ultrasonic irradiation ^26^ have been reported.

Acoustic cavitation is a physical phenomenon that helps chemical reactions under ultrasound irradiation. Ultrasound has been known as significant for green and remarkable synthetic methods ^27–29^. The ultrasound approach reduces times, increases yields of products by creating the activation energy in micro surroundings ^30,31^. This phenomenon is generally contained the construction, growth, and transient implosive collapse of the gas and vapour filled microbubbles. The physical and chemical effects of cavitation are exciting for various applications ^32^. This method indicates bubble-sphere interaction on a microscale. The presence of suspended spherical particles near to substances of reactant could potentially have an important effect on bubble dynamics ^33,34^. Precipitate implosion of these bubbles in the liquids creates localized hot spots with very short lifetimes. The hot spot has an equivalent temperature of 5000 °C and pressure of about 2000 atmospheres can achieve upon the destruction of the bubble ^35^ without any significant change in the reaction medium (in terms of pressure and temperature) ^36^. The physical properties of the catalyst, including good thermal, hydrothermal, and high mechanical stabilities are very important in the choice of catalyst.

Catalyst scientists and nanotechnology have helped a lot in this regard. In our search of nanocatalysts, we used dendritic silica nanospheres (KCC-1) as the catalyst ^37–39^. Fibrous nano-silica sphere (KCC-1) compared to MCM-41 and SBA-15-supported catalysts, shows excellent physical properties, including a high surface area, a fibrous surface morphology, good thermal/hydrothermal properties and high mechanical stability. KCC-1 can be used as catalyst support, sorbent or carrier. Due to the unique properties of silica is used in various organic reactions ^40–43^, drug delivery systems and biomedical applications ^44^, optoelectronic devices ^45^, modern industries ^46–49^, gas capture, solar energy harvesting ^50,51^ and many others.

In the synthesis of fibrous nano-silica (KCC-1), we can control particle size, fiber density, surface area and pore volume of KCC-1 and tune by changing various reaction parameters, such as the concentrations of urea, CTAB, 1-pentanol, reaction time, temperature, solvent ratio, and even outside stirring time ^52^. Furthermore, it is the fibrous morphology of KCC-1 that produces better accessibility of the active sites for enhanced catalytic activities and recovery efficiencies ^52^. As well as the mechanical and thermal stability of KCC-1 provides the better heterogeneous catalyst for ultrasonic irradiation conditions. In this regard, we chose an easy, fast and green method for the synthesis of 2-amino chromenes with modified dendritic silica nanosphere (KCC-1@NH_2_) by the ultrasonic route. Also, we managed to synthesize some new derivatives of 2-Amino-4H-Chromenes.

## Results and discussion

### Structural analysis of the KCC-1@NH_2_ nanocatalyst

**Scheme 1:**
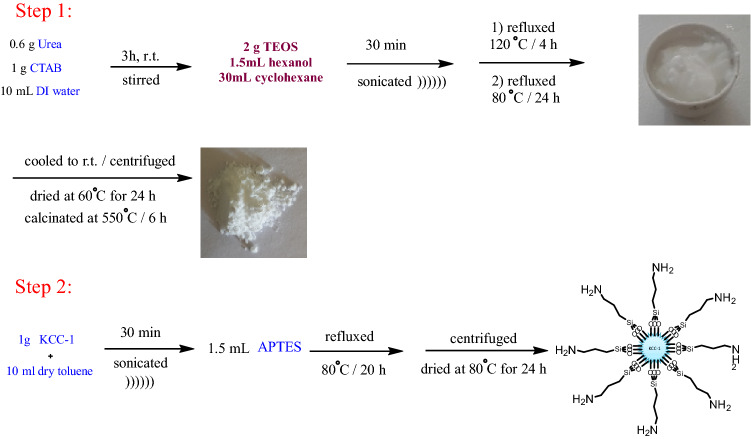
The preparation of nanosphere KCC-1@NH_2_.

In this study, first fibrous nanosilica spheres was prepared with the methods was reported by Bayal et al. ^52^. In the second stage, a NH_2_ shell using APTES (aminopropyltriethoxysilane) was coated on the nanosilica core. The KCC-1@NH_2_ nanocatalyst was as an efficient basic catalyst for the preparation of 2-Amino-4H-chromenes (Scheme 1).

**Figure 2:**
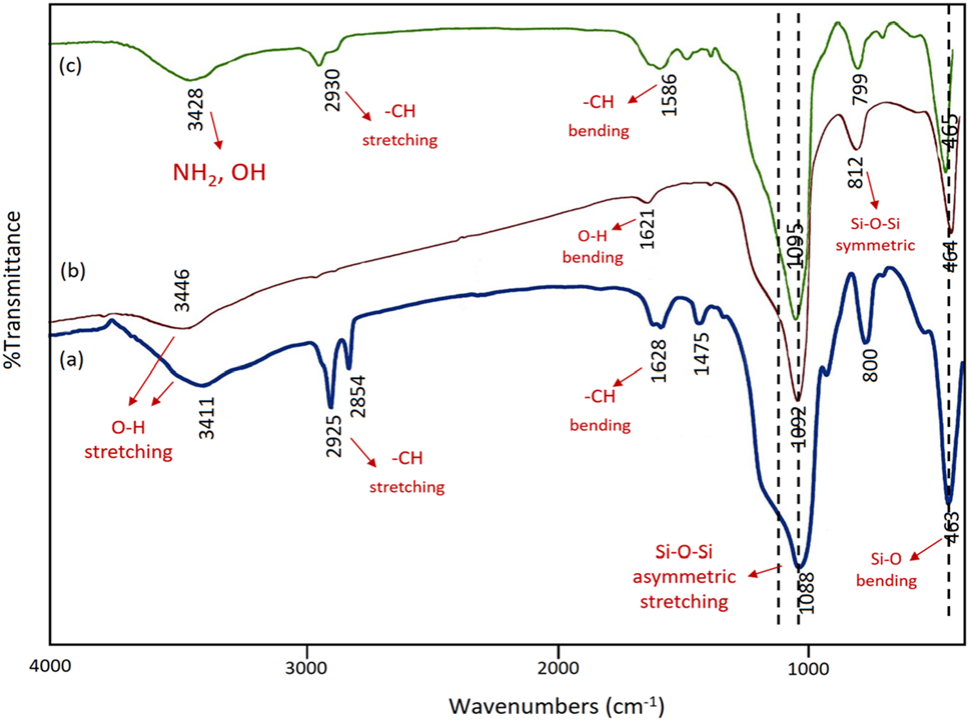
The FT-IR spectra of (a) KCC-1 before calcination, (b) KCC-1 after calcination and (c) KCC-1@NH_2_.

The IR patterns of different stages of nanosilica preparation are showed in Fig. 2. The characteristic peaks of the silica-based materials could be observed in the range of 1092 to 1150 cm^−1^ representing the Si–O–Si asymmetric stretching vibration while a Si–O–Si peak is observed at 812 cm^−1^, which represents the symmetric stretching vibration. In addition, the peaks at around 463 cm^−1^, 1621 cm^−1^ and 3446 cm^−1^ can be assigned to the Si–O bending vibration, O–H bending and stretching vibration, respectively (Fig. 2b). As shown in Fig. 2a,b compared to KCC-1 before calcination, the –CH_2_ and –CH_3_ new peaks were removed after calcination. In addition, the peaks at around 2930 cm^−1^ and 1586 cm^−1^ can be assigned to the –CH stretching and bending vibration derived from the CH_2_ groups of the alkyl chains, respectively (Fig. 2c). These FT-IR spectral features indicated the successful functionalization of APTES over KCC-1.

**Figure 3:**
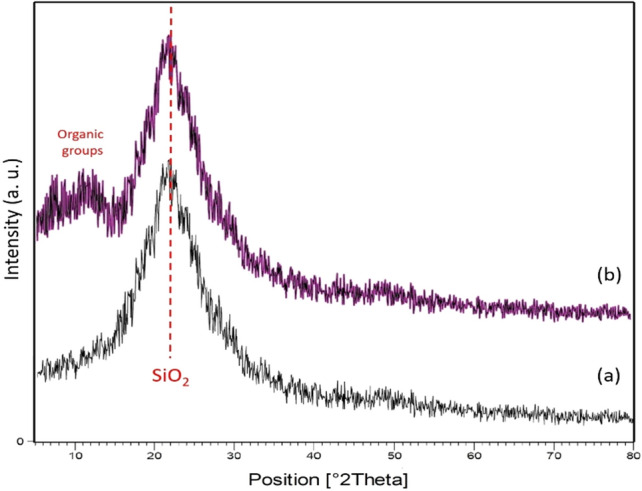
The XRD pattern of nanosphere (a) KCC-1 and (b) KCC-1@NH_2_.

The XRD pattern of nanosilica spheres KCC-1 and KCC-1@NH_2_ is depicted in Fig. 3a,b, respectively. Figure 3b reveals high phase purity of the nanocatalyst and has a perfect agreement with the reported XRD pattern for nanosilica spheres (KCC-1@NH_2_). The broad peak between 20° and 30° in Fig. 3b, corresponds to amorphous silica ^53^. The XRD pattern of KCC-1@NH_2_ includes peaks from SiO_2_ and organic layer on this catalyst. The average crystalline size of the nanocatalyst was calculated to be 8 nm that was obtained from FWHM Scherrer’s formula.

**Figure 4:**
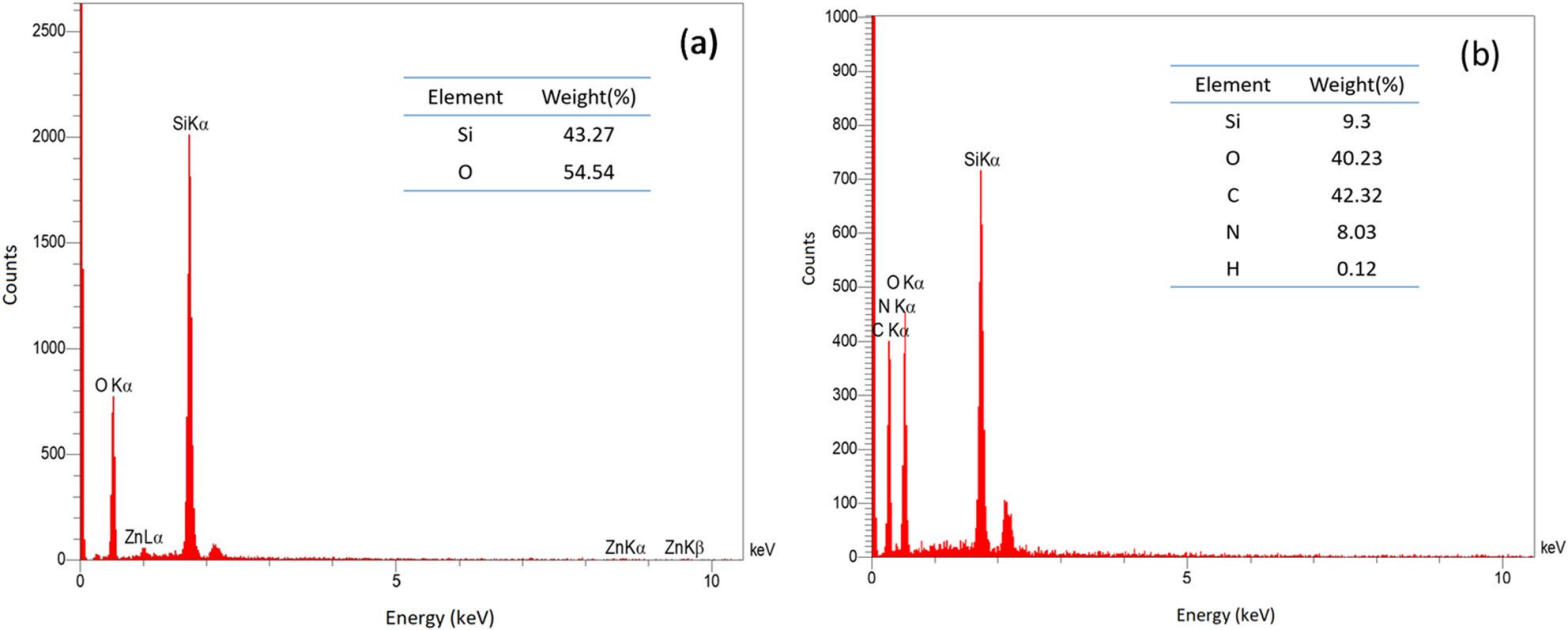
The EDX spectrum of nanosphere (a) KCC-1 and (b) KCC-1@NH_2_.

The elemental compositions of the fibrous nanosilica spheres (KCC-1@NH_2_) were demonstrated by Energy Dispersive Spectroscopy (EDX). According to the outcome data, all expected elements including silicon, oxygen, carbon and nitrogen were approved (Fig. 4).

**Figure 5:**
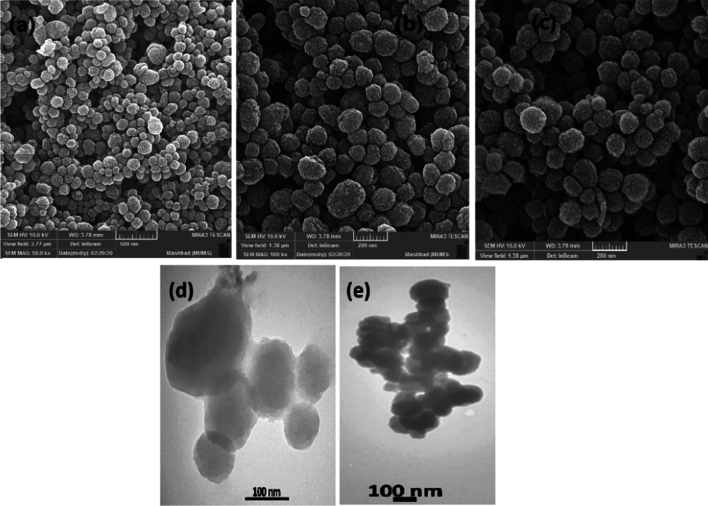
The SEM image of nanosphere KCC-1@NH_2_ (**a**) KCC-1, (**b**) KCC-1@NH_2_ before use, (**c**) after reuse of six times, and (**d,e**) TEM images of KCC-1@NH_2_.

By the scanning electron microscopy (SEM) image, morphology, and particle size of fibrous nanosilica spheres (KCC-1@NH_2_) is confirmed (Fig. 5). Scanning electron microscopy (SEM) images (Fig. 5) indicate that the material consists of colloidal spheres of uniform size with diameters that range from 50 to 190 nm and the nanoparticles show good dispersity with spherical morphology. The SEM image of the reused catalyst for six runs is shown in Fig. 5b. This image is confirmed the high stability of the reused nanocatalyst after five runs.

The morphological features of the sample were characterized by Transmission electron microscopy. For the preparation of samples for this type of observation, First, we put a very small amount of synthesized powder in a glass containing a suitable dispersant (ethanol, acetone, distilled water, etc.) of that sample. The dilute aqueous solution of sample was sonicated for 15 min by Misonix sonicator (Misonix-S3000, USA) then One drop of the sample was dropped onto formvar carbon film on copper grid 300 mesh (EMS-USA) and dried thoroughly at room temperature ^54,55^. The sample was observed by transmission electron microscopy (TEM, model Zeiss-EM10C Company) at accelerating voltage 100 kV. Close inspection of these images reveals that the material possesses dendrimeric fibers (for example angled with thicknesses of 8–10 nm) arranged in three dimensions to form spheres, which can allow easy access to the available high surface area. Further structural characterization of synthesized silica nanosphere performed by high-resolution transmission electron microscopy (HRTEM) reveals well-defined and ordered fibers coming out from the centre of the particles and distributed uniformly in all directions. The TEM image of the KCC-1@NH_2_ nanocatalyst (Fig. 5d,e) demonstrates that wrinkled fibers grow out from the centre of the spheres and are arranged radially in three dimensions. The TEM image provides more exact information about the particle size and morphology of the nanomaterial. The TEM image tells the spherical shape of the nanosilica, with an average size of 97 nm, which shows near agreement with the value determined via SEM. The FE SEM and TEM image indicates that the entire sphere is solid and composed of spherical.

**Figure 6:**
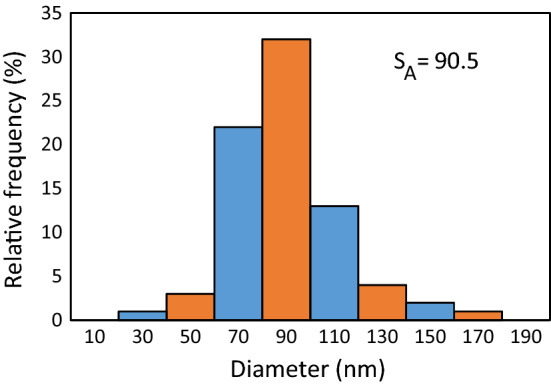
The size distribution of particles.

Also, the size distribution of particles was provided using SEM image via Digimizer Image Analysis Software (DIAS) was shown in Fig. 6.

It should be noted, the morphology of the silica can also be affected by the precursor materials, the hydrolysing reagent (in this case urea), urea concentration and the solvents. Results indicate that the key to the fibrous morphology as well as particle size is the control on the speed of the TEOS hydrolysis by urea ^38^.

**Figure 7:**
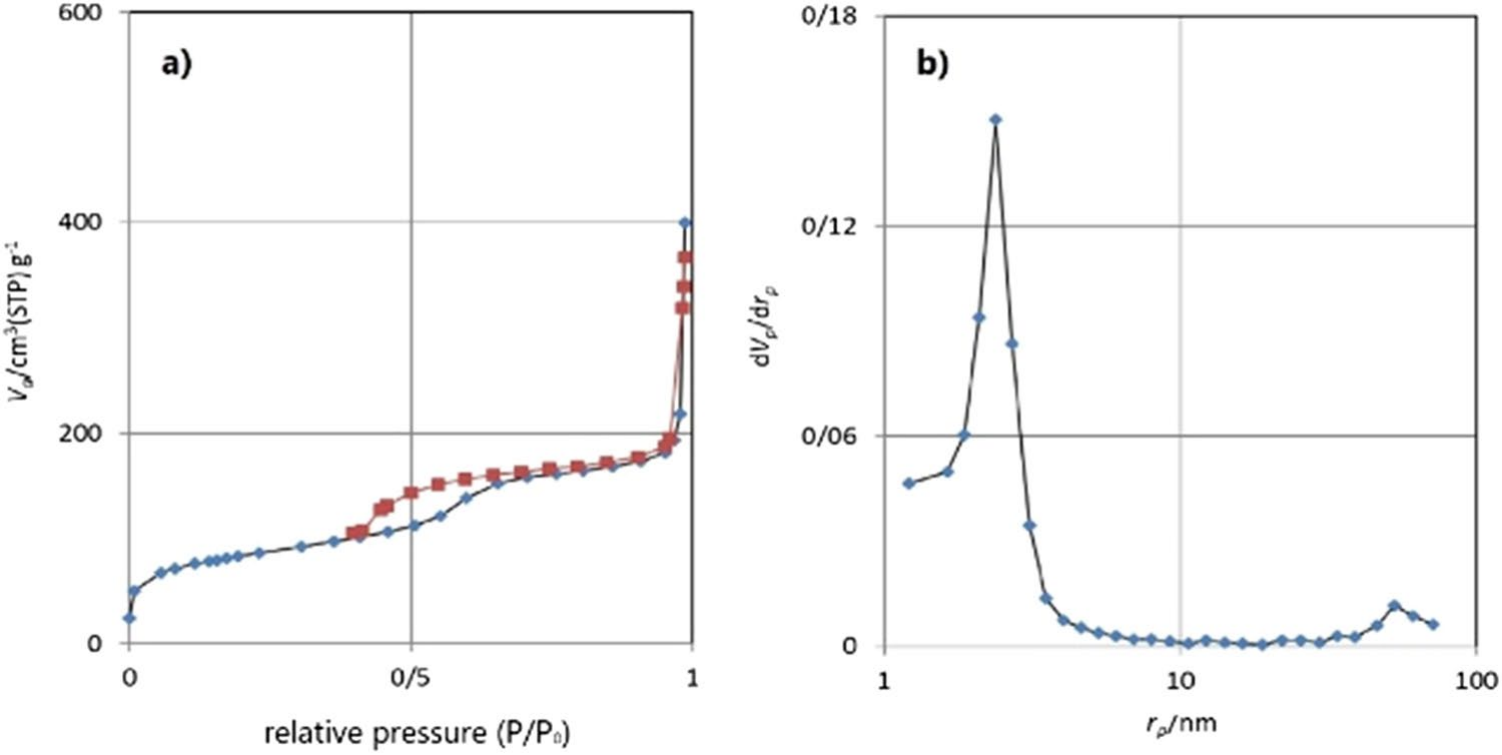
The BET and BJH images of KCC-1@NH_2_.

Nitrogen adsorption–desorption isotherms analysis and BJH pore size distributions are done to evaluate the surface and structure properties of KCC-1@NH_2_ (Fig. 7). According to the International Union of Pure and Applied Chemistry (IUPAC) classification, this catalyst indicated characteristic type IV curve, which is consistent with literature reports on standard fibrous silica spheres. H2 type hysteresis loop in the relative pressure ranges from 0.4 to 1.00, is attributed to mesoporous materials. For KCC-1@NH_2_, the BET surface areas were 297 m^2^ g^−1^; pore diameters were 8.32 nm; and pore volumes 0.62 cm^3^ g^−1^, respectively.

**Figure 8:**
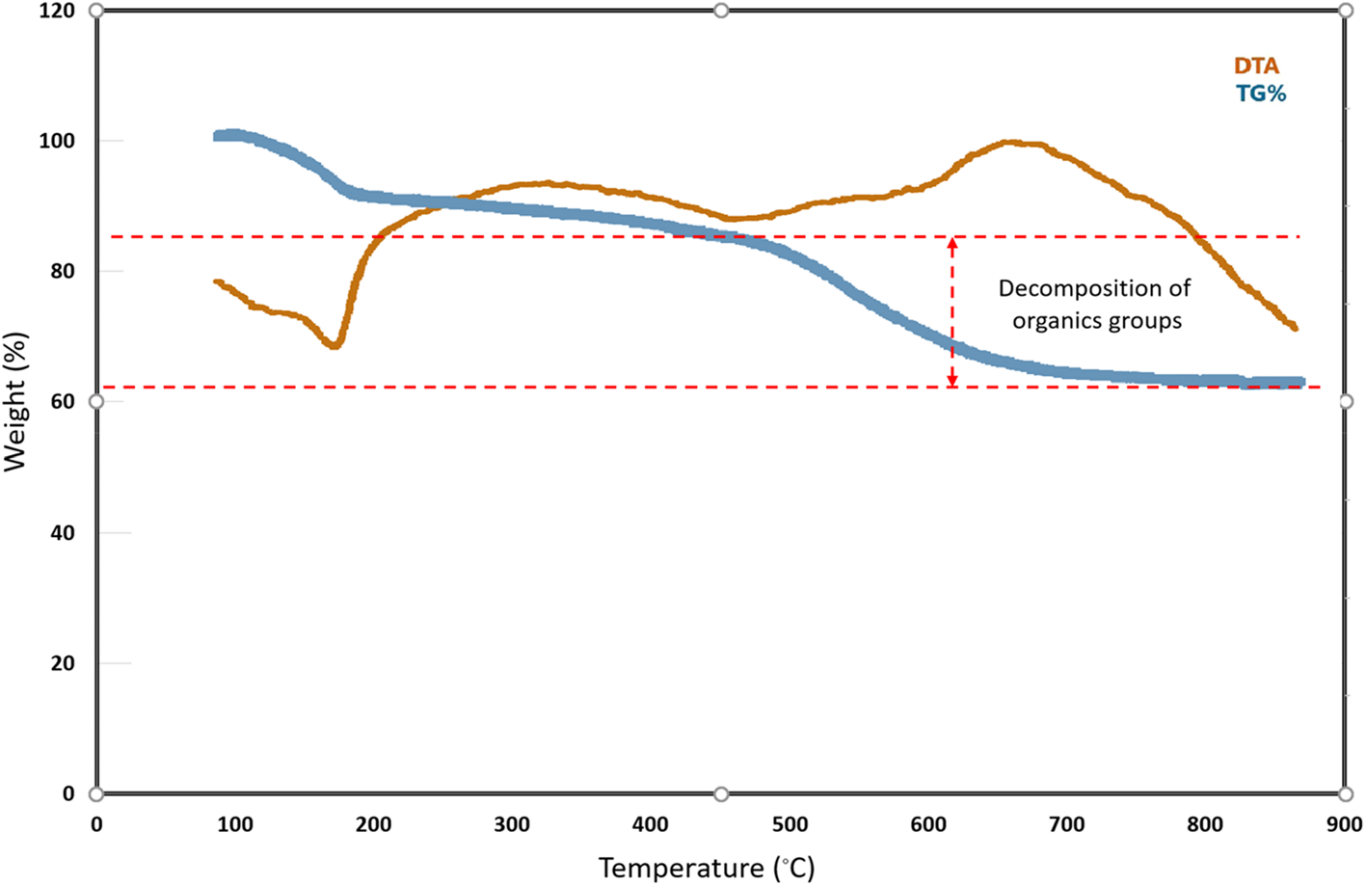
The TGA curves of KCC-1@NH_2_.

The thermal behaviour of nanosilica spheres (KCC-1@NH_2_) is shown in Fig. 8. The TG profile exhibits two steps of weight loss. The initial mass loss of 8% accrued with an endothermic peak in DTA curve is revealed in the temperature range of 80–110 °C. It can be related to the release of physically absorbed water or solvent on the surface of the KCC-1@NH_2_ and other raw materials. The second mass loss of 28% in a wide temperature range of 460–640 °C, it corresponds mainly to the thermal decomposition of the organics group. The results of the thermal analysis expressed that the thermal stability of nanocatalyst is up to near 500 °C.

### Synthesis of 2-amino-4H-chromenes catalyzed by nanosilica KCC-1@NH_2_

**Scheme 2:**
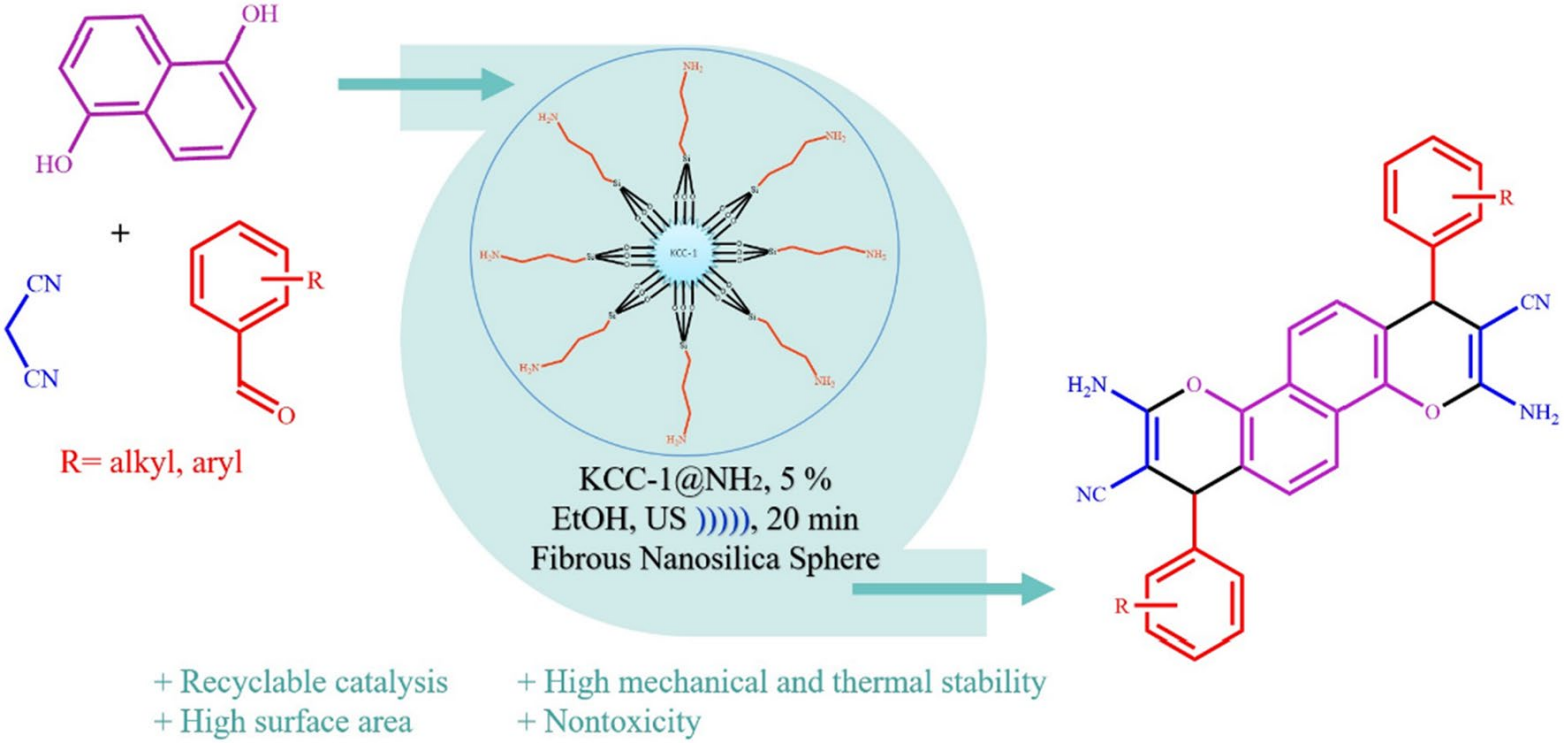
The use of KCC-1@NH_2_ in preparation of 2-amino-4H-chromenes.

Chromenes derivatives have been prepared from the Knoevenagel condensation of 1,5-naphtalenediol, malononitrile and aromatic aldehydes compounds catalyzed by nanosilica at 20 kHz frequency and 80 W power for an appropriate time under ultrasound irradiation as drawn in Scheme 2.

The effect of experimental factors comprising type and amount of catalyst, different powers of ultrasound irradiation, different solvents and effects of different donor or withdrawing substitutions of aldehydes were investigated to find the best condition for this reaction and the results are listed in Tables For this purpose, the reaction between 4-chlorobenzaldehyde (1.0 mmol), malononitriles (1.0 mmol), and 1,5-dinaphtol (0.5 mmol) as substrates by prepared nanosphere as a catalyst under ultrasonic conditions was selected as the model reaction. At first, the optimum amount of catalyst was investigated in ethanol solvent (Table 1).

**Table 1:** Optimization of reaction condition for the formation of 2-amino-4H-chromenes.

Entry	Solvent	Catalyst (g)	Time (min)	Yield^a^%
1	DMF	Nano KCC-1@NH_2_ (0.05)	20	80
2	CH_3_CN	Nano KCC-1@NH_2_ (0.05)	20	80
3	H_2_O	Nano KCC-1@NH_2_ (0.05)	20	90
4	THF	Nano KCC-1@NH_2_ (0.05)	20	60
5	EtOH	Nano KCC-1@NH_2_ (0.05)	20	96
6	EtOH	Nano KCC-1@NH_2_ (0.08)	20	96
7	EtOH	Nano KCC-1@NH_2_ (0.03)	20	90
8	EtOH	TEA	20	85
9	EtOH	Piperidine	20	85
10	EtOH (reflux)	Nano KCC-1@NH_2_ (0.05)	180	70

Reactions conditions: p-chloro benzaldehyde (1 mmol), 1,5-naphtalendiol (0.05 mmol), malononitrile (1 mmol).

Ultrasonic irradiation (80 W).

^a^Isolated yield.

The effect of experimental factors comprising type and amount of catalyst, different powers of ultrasound irradiation, different solvents and effects of different donor or withdrawing substitutions of aldehydes were investigated to find the best condition for this reaction and the results are listed in Tables For this purpose, the reaction between 4-chlorobenzaldehyde (1.0 mmol), malononitriles (1.0 mmol), and 1,5-dinaphtol (0.5 mmol) as substrates by prepared nanosphere as a catalyst under ultrasonic conditions was selected as the model reaction. At first, the optimum amount of catalyst was investigated in ethanol solvent (Table 1).

With reference to the results shown in Table 1, the optimized quantity of nanocatalyst for this synthesis is 0.05g (Table 1, entry 5). In an effort to obtain better yields and the most effective solvent, various solvents were used for the synthesis of chromenes. The examination of solvent was demonstrated that ethanol as protic solvent is the best condition for the Knoevenagel condensation of benzaldehydes and malononitrile compounds (Table 1, entry 5). Comparison of this entry with entries 8–9 of Table 1 (various catalysts containing piperidine and NEt_3_) reveals that the nanocatalyst is the most efficient catalyst for the sonochemical synthesis of 2-amino chromenes.

Evaluation of thermal and ultrasound conditions shows that the ultrasonic approach is very effective for this synthesis is presented in Table 1. When the 2-aminochromenes derivatives were synthesized under the heating method (entry 10, Table 1), they were produced in lower yields at higher reaction times, but performing these reactions under sonication conditions created excellent yields of 2-Aminochromenes at short times. Therefore, because of its basic green chemistry conception, the shock wave and microjet generated by the cavitation, this method is more environmentally benign. During the ultrasonic irradiation, KCC-1@NH_2_ nanocatalyst like a wall for the transmission of the bubble, is dispersed in the reaction and affords more sites for the generation of the number of micro-bubbles. Increasing of micro-cavities may advance the helpfulness of the ultrasound approach to the formation of 2-Aminochromenes ^41–44^.

In continues, to detect the suitable power of ultrasonic irradiation for this reaction, it was tested under different powers of ultrasound irradiation as shown in Table 2. In the end, this reaction is effectively proceeded by 0.05 g of KCC-1@NH_2_ nanocatalyst with the power of 80 W of ultrasonic irradiation. Really in ultrasound irradiation the number of active cavitation bubbles and size of the individual bubbles is to increase. As a result, collapse temperature was increased and accelerated the synthesis of 2-amino-chromenes derivatives reaction. Various substituted 2-Aminochromenes were prepared by nanocatalyst using the obtained optimized condition (Table 3). The results were indicated that aromatic aldehydes with electron-withdrawing groups reacted much more faster compared to those with electro-donating groups.

**Table 2:** Optimization of reaction condition for the formation of 2-amino-4H-chromenes.

Entry	Power (W)	Time (min)	Yield^a^ (%)
1	50	20	45
2	60	20	65
3	70	20	82
4	80	20	96
5	90	20	96

Reactions conditions: p-chloro benzaldehyde (1 mmol), malononitrile (1 mmol), 1,5-dinaphtol compound (0.5 mmol), nano KCC-1@NH_2_ (5%).

^a^Isolated yields.

**Table 3:** Synthesis of 2-amino-4H-chromenes using nanosphere KCC-1@NH_2_.


Entry	Product^a^	Time (min)	Yield^b^ (%)	M.P. (°C)^c^
1		30	85	> 300
2		30	80	> 300
3		30	84	315–320
4		30	82	310–320
5		20	96	> 300
6		35	80	262–270
7		25	92	310–315^56^
8		30	82	> 300^56^
9		25	90	295–300^57^
10		20	96	300–310^58,59^
11		20	95	320–325^57^
12		20	95	300–310^57^
13		25	93	> 300^57^
14		30	82	251–258^57^
15		35	80	265–274^57^

Reactions conditions: benzaldehyde (1 mmol), malononitrile (1 mmol), 1,5-dinaphtol compound (0.5 mmol), nano KCC-1@NH_2_ (5%) under ultrasonic irradiation (80 W).

^a^All products were characterized from their spectroscopic IR, ^1^H NMR.

^b^Isolated yield.

^c^Literature references.

**Scheme 3:**
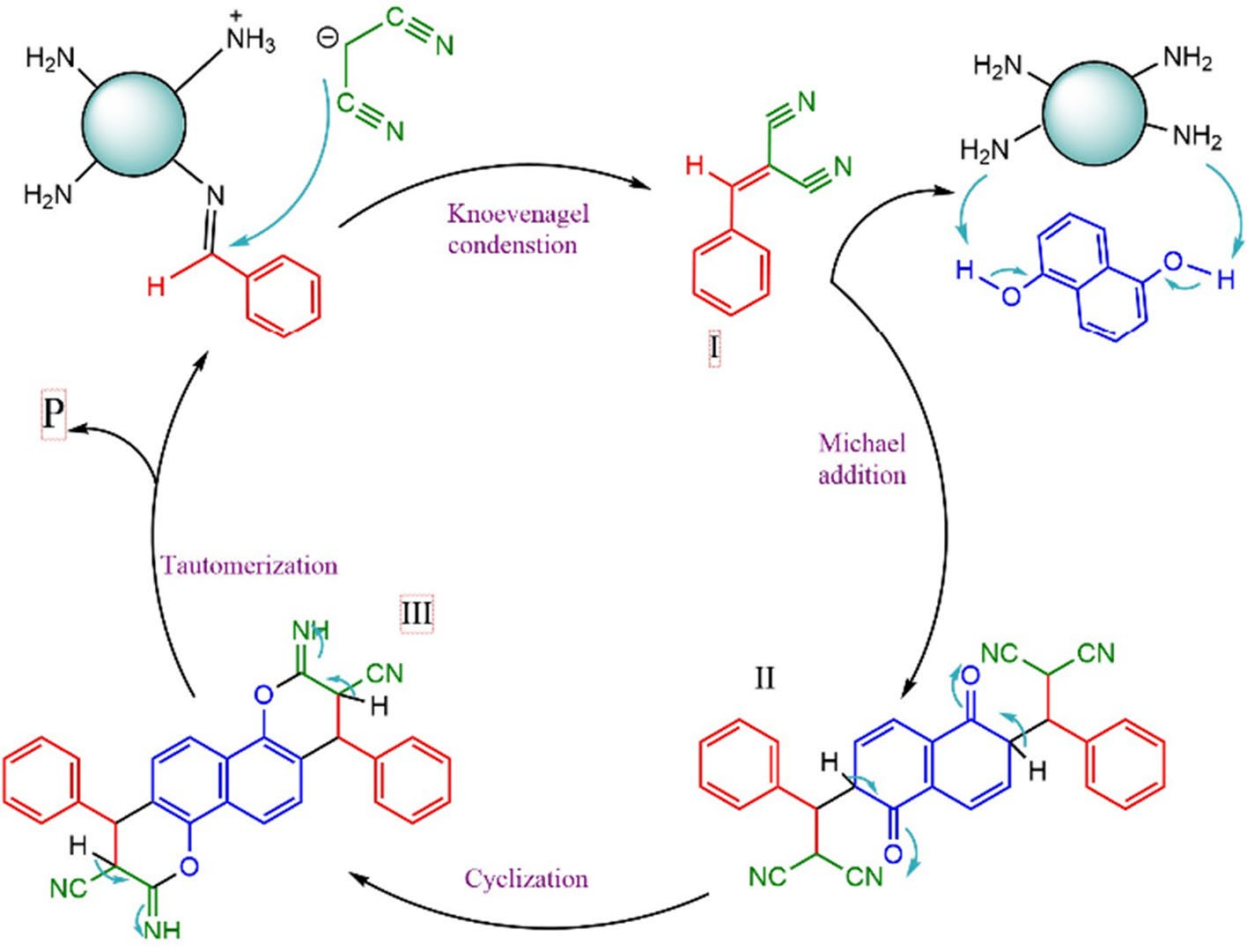
The probable mechanism of synthesis 4a in the presence of nanosilica spheres KCC-1@NH_2_.

A rational mechanism for the preparation of 2-aminochromens under ultrasonic irradiation by the KCC-1@NH_2_ nanocatalyst is illustrated in Scheme 3. At first, a imine was formed between the carbonyl group of aldehyde and the NH_2_ group of nanocatalysts ^60,61^. Also, acidic hydrogen of malononitrile can be removed by nanocatalyst. Afterward, by a Knoevenagel condensation, active methylene of malononitrile attacked to imine and affords to intermediate I after removing one molecule of H_2_O. Then, 1,5-dinaphthol-activated by catalyst-from β-position attacks to the cyanoolefin compound (I) to give II. Finally, further aromatization and intramolecular cyclization of II gives III which is converted to the corresponded product. These steps are efficiently offered on the cavitation effect of ultrasound irradiation and also by the high nanosphere surface. Based on this mechanism, it is highly probable that the carbonyl groups of aldehydes and malononitrile compounds have been activated, by the coordination of carbonyl oxygen and KCC-1@NH_2_ nanospheres. Thus KCC-1@NH_2_ nanospheres activated of methylene of malononitrile and carbonyl groups. Due to its high surface area increase the rate and yield of the reaction. In addition, the catalyst used is easily filtrated and reused without any noticeable loss of activity after at least five times (Scheme 3).

### Reusability of KCC-1@NH_2_ nanocatalyst

**Figure 9:**
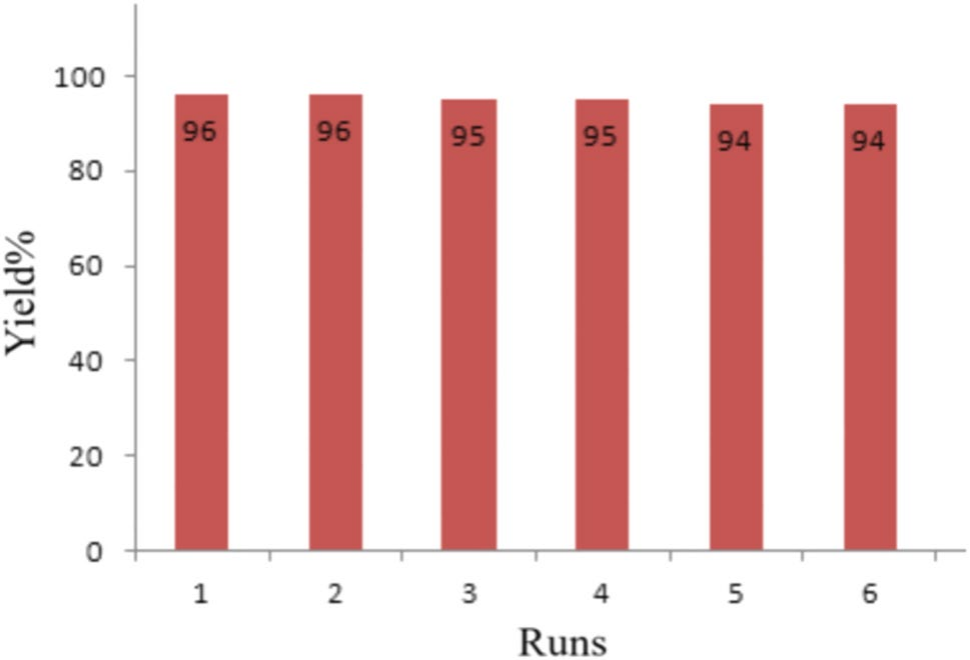
Recovery of nanosphere KCC-1@NH_2_.

Reusability and recoverability of Nanosilica spheres (KCC-1@NH_2_) are known as one of the most important properties of the catalyst under ultrasonic conditions. After the completion of reaction, 5 mL of acetone was added to the reaction mixture. The product solved in acetone and nanosilica was recycled via filtration. The reusability of our catalyst was tested for the model reaction, and it was found that product yields lessened only nanosilica spheres (KCC-1@NH_2_) are recoverable without a considerable loss of catalytic activity (Fig. 9). It was very important to us that the catalyst was stable in ultrasonic irradiation conditions. Accordingly, we investigated the morphology and particle size of the nanocatalyst before use and after reuse six times in reaction by SEM image as presented in Fig. 5b,c. According to the figures, the morphology of the nanoparticles stayed unchanged. We believe this is also the possible reason for the extreme stability of the fibrous nanosilica spheres for ultrasonic irradiation conditions.

In Table 4 was showed different reports in the literature for the synthesis of 2-amino chromenes. Table 4 represents the differences between their results (entries 1–5) and the results of the present research (entry 6). As can be seen the proposed method in this work is the best condition for the synthesis of 2-amino chromenes derivatives. The properties such as mild reaction condition, high yields of 2-amino chromenes, easy recovery of the nanosilica by filtration, reusability of the catalyst for 6 times without significant loss of catalytic performance, short reaction times and environmentally benign of this method makes better than other previous methods. The main drawback of other procedures is a non-reusable catalyst, long reaction time, difficulty in separation of catalyst from the reaction mixture and low efficiency.

**Table 4:** Comparison the results of synthesis of 2-Amino-chromens through the Knoevenagel condensation in this research and various reports in the literature.

Entry	Catalyst^a^	Solvent	Reaction condition	Time (min)	Yield (%)^b^
1	Nano ZnO (0.5 mmol) ^60^	H_2_O	Thermal/80 °C	180	70
2	KF/Al_2_O_3_ (0.5 g) ^57^	EtOH	Thermal/80 °C	300–360	93
3	TEA (2–3 drops) ^18^	–	Microwave/300 W	5–6	82
4	TEA (0.5 mL) ^18^	EtOH	Thermal/80 °C	10	82
5	Piperidine (0.1 mL) ^56^	EtOH	Thermal/80 °C	30	65
6	Nano KCC-1@NH_2_ (0.05 g)	EtOH	US/80 W	20	96

^a^Literature references.

^b^Isolated yield.

## Experimental section

### Substances and method

Cetyltrimethylammonium bromide (CTAB) ([(C_16_H_33_)N(CH_3_)_3_]Br), Urea, Tetraethyl orthosilicate (Si(OC_2_H_5_)_4_, Merck, > 98%), Cyclohexane, hexanol, (3-Aminopropyl) triethoxysilane (APTES), dried Toluene (C_7_H_8_, Merck, > 99.8%), absolute Ethanol (C_2_H_5_OH, Merck, > 99.9%), Malononitril, 1,5-dihydroxynaphthalene, derivatives Aldehyde were acquired from Merck and Sigma-Aldrich Company. They were used immediately without further filtration and distilled water was used throughout the test.

In this reaction, we were applied the ultrasonic irradiation using a multiwave ultrasonic generator (Sonicator 3200; Bandelin, MS 73, Germany), armed by a converter/transducer and titanium oscillator (horn), 12.5 mm diameter, operating at 20 kHz with a maximum power output of 200 W. The ultrasonic generator automatically harmonized the power level. Melting points of synthesized products were determined by Electrothermal 9200. IR spectra of synthesized products and different stages of catalyst fabrication were noted by means of FT-IR Magna spectrometer 550 Nicolet using KBr plates. ^13^C NMR and ^1^H NMR spectra were reached in DMSO-d_6_ as a solvent on Bruker Avance-400 MHz spectrometers in TMS as an internal standard. The microscopic morphology of the nanoparticles was visualized by the morphological features of the sample were investigated with a Zeiss (EM10C-Germany) transmission electron microscope (TEM) operating at 100 kV and field emission scanning electron microscopy (FE SEM) (MIRA 3 TESCAN). Energy-dispersive X-ray spectroscopy (EDX) of the nanoparticles was imagined by a Sigma ZEISS, Oxford Instruments Field Emission. For surface area measurement was used of Brunauer Emmett Teller (BET) method. It was determined by nitrogen adsorption amount using a mechanized gas adsorption analyzer (Tristar 3000, Micromeritics). Powder XRD of KCC nanoparticles was achieved by a Philips diffractometer of X’pert Company. Thermogravimetric and differential thermal analysis (TGA-DTA) were obtained from a Bahr STA-503 instrument in the air at a heating rate of 10 °C min^−1^. The purity determination of the substrates and reaction monitoring was accomplished by TLC on silica gel polygram SILG/UV 254 plates (from Merck Company).

### Preparation of fibrous nanosilica spheres (KCC-1)

Bayal and co-workers reported the methods of synthesizing of KCC-1 ^52^. Briefly, 1 g CTAB was added to 10 mL deionized water and after 0.6 g urea was added to the flask, the mixture was stirred for about 3 h at room temperature. Then, the mixture of 2 g TEOS, 1.5 mL hexanol and 30 mL cyclohexane was added to the flask and sonicated for 30 min. Later, the mixture was refluxed at 120 °C for 4 h and afterward refluxed at 80 °C for 24 h. Then, the mixture was cooled to room temperature and centrifuged to collect the KCC-1 as white Sediment. The collected KCC-1 was washed several times with water and ethanol and dried at 60 °C for 24 h. Finally, KCC-1 was calcinated at 550 °C for 6 h to remove the CTAB as a templating agent. For this mechanism, urea was added to hydrolyse the TEOS to produce negatively charged (SiO_4_)_4_^−^ silicate. Using of CTAB persuades the silicate molecules to form self-assembled linear structures where the CTAB helps to the aggregating of the silicates ^37,62^.

### Preparation of KCC-1@NH_2_

To functionalize the KCC-1 surface with NH_2_ moieties, 0.02 g of KCC-1 was dispersed on 1.2 mL dried toluene and sonicated for 30 min. Then 50 μL 3-aminopropyltriethoxysilane (APTES) was added to the mixture and refluxed for 20 h at 80 °C. Then the mixture was separated and washed with toluene several times and dried at 80 °C for at least 24 h ^63^.

### General procedure for the preparation of 2-amino-4H-chromenes using functionalized fibrous nanosilica sphere (KCC-1@NH_2_) under ultrasonic irradiation

A mixture of 1,5-naphtalenediol (1 mmol), malononitrile (2 mmol) and aromatic aldehydes (2 mmol) and fibrous nanosilica sphere (KCC-1@NH_2_) (0.05 g) as a catalyst in ethanol (5 mL) was sonicated at 20 kHz frequency and 80 W power for required times. The reaction was monitored by TLC. After completion of the reaction, the reaction mixture dissolved with acetone. Then, nanocatalyst was filtered and washed with toluene, dried and re-used for a successive run under the same reaction conditions. Evaporation of the solvent of the residual solution under reduced pressure gave a crude product. The solid product was recrystallized with EtOH to get pure product. The products were characterized based on ^1^H-NMR, FT-IR, and melting point analysis, and the spectral data of the synthesized compounds were compared with authentic samples. The Spectra data of new compounds are presented:

### Spectral data

3,9-Diamino-1,7-bis(3-hydroxyphenyl)-1,7-dihydrochromeno[8,7-h]chromene-2,8-dicarbonitrile (**1**): 90%, Yellow solid, m.p. > 300 °C (decomp.), IR (KBr) ν (cm^−1^): 3444 (NH_2_), 3301 (NH_2_,OH), 2192(CN), 1652 (NH_2_ bending), 1596, 1455, 1386, 1280, 1246, 1187, 1081 (C–O), 886, 761; ^1^H NMR (DMSO-d6, 400 MHz) $\delta$ (ppm): 4.78 (s, 2H, CH_benzyl_), 6.67–6.69 (m, 4H, H_aromatic_), 7.07–7.10 (m, 4H, H_aromatic_), 7.13 (br.s, 4H, NH_2_), 7.22–7.24 (m, 2H, H_aromatic_), 7.86–7.88 (dd, J = 8.4 Hz, 2H, H_aromatic_), 9.35 (s, 2H, OH) (see SI, Figs. S1–S3).

3,9-Diamino-1,7-bis(2-hydroxyphenyl)-1,7-dihydrochromeno[8,7-h]chromene-2,8-dicarbonitrile (**2**): 85%, Yellow solid, m.p. > 300 °C (decomp.), IR (KBr) ν (cm^−1^): 3467 (NH_2_), 3332 (NH_2_, OH), 3196, 2192(CN), 1650 (NH_2_ bending), 1595, 1461, 1383, 1280, 1187, 1080 (C–O), 889, 799; ^1^H NMR (DMSO-d6, 400 MHz) $\delta$ (ppm): 5.11 (s, 2H, CH_benzyl_), 6.91–6.93 (d, J = 7.2 Hz, 2H, H_aromatic_), 6.98–7.01 (d, J = 8.8 Hz, 2H, H_aromatic_), 7.27 (s, 4H, NH_2_), 7.39–7.43 (dd, J = 8 Hz, 2H, H_aromatic_), 7.66–7.68 (d, J = 8.4 Hz, 2H, H_aromatic_), 7.77–7.80 (d, J = 8.8 Hz, 2H, H_aromatic_), 8.18–8.20 (d, J = 8.4 Hz, 2H, H_aromatic_), 10.30 (s, 2H, OH) (see SI, Figs. S4–S6).

3,9-Diamino-1,7-bis(4-isopropylphenyl)-1,7-dihydrochromeno[8,7-h]chromene-2,8-dicarbonitrile (**3**): 82%, Yellow solid, m.p = 315–320 °C (decomp.), IR (KBr) ν (cm^−1^): 3492 (NH_2_), 3379 (NH_2_), 2198(CN), 1651 (NH_2_ bending), 1596, 1455, 1386, 1272, 1232, 1187, 1086 (C-O), 755; ^1^H NMR (DMSO-d6, 400 MHz) $\delta$ (ppm): 1.14 (s, 6H, CH_3_), 1.16 (s, 6H, CH_3_), 2.80–2.83 (m, 2H, CH) 4.84 (s, 2H, CH_benzyl_), 7.12 (br.s, 4H, NH_2_), 7.13–7.27 (m, 10H, H_aromatic_), 7.85–7.88 (dd, J = 8.4 Hz, 2H, H_aromatic_) (see SI, Figs. S7–S9).

3,9-Diamino-1,7-bis(4-methylphenyl)-1,7-dihydrochromeno[8,7-h]chromene-2,8-dicarbonitrile (**4**): 85%, Yellow solid, m.p = 310–320 °C (decomp.), IR (KBr) ν (cm^−1^): 3454 (NH_2_), 3325 (NH_2_), 3202, 2922, 2196(CN), 1659 (NH_2_ bending), 1596, 1500, 1388, 1282, 1238, 1187, 1084 (C–O), 854, 763; ^1^H NMR (DMSO-d6, 400 MHz) $\delta$ (ppm): 2.07 (s, 6H, CH_3_), 5.40 (s, 2H, CH_benzyl_), 7.09–7.13 (m, 2H, H_aromatic_), 7.22–7.29 (m, 8H, H_aromatic_ and NH_2_), 7.37–7.40 (m, 2H, H_aromatic_), 7.61–7.63 (dd, J = 8 Hz, 2H, H_aromatic_), 7.86–7.88 (dd, J = 8.8 Hz, 2H, H_aromatic_) (see SI, Figs. S10–S12).

3,9-Diamino-1,7-bis(4-nitrophenyl)-1,7-dihydrochromeno[8,7-h]chromene-2,8 dicarbonitrile (**5**): 94%, Yellow solid, m.p. > 300 °C (decomp.), IR (KBr) ν (cm^−1^): 3441 (NH_2_), 3336 (NH_2_), 3196, 2190(CN), 1655 (NH_2_ bending), 1597, 1527, 1387, 1350, 1281, 1187, 1082 (C–O), 800, 728; ^1^H NMR (DMSO-d6, 400 MHz) $\delta$ (ppm): 5.21 (s, 2H, CH_benzyl_), 7.28–7.30 (m, 4H, H_aromatic_), 7.34 (br.s, 4H, NH_2_), 7.60–7.73 (m, 6H, H_aromatic_), 7.90–7.92 (d, J = 8 Hz, 2H, H_aromatic_), 8.12 (br.s, 2H, H_aromatic_) (see SI, Figs. S13–S15).

3,9-Diamino-1,7-bis(2,3-dimethoxyphenyl)-1,7-dihydrochromeno[8,7-h]chromene-2,8-dicarbonitrile (**6**): 83%, Yellow solid, m.p > 300 °C (decomp.), IR (KBr) ν (cm^−1^): 3430 (NH_2_), 3315 (NH_2_), 2195 (CN), 1654 (NH_2_ bending), 1599, 1477, 1386, 1284, 1077 (C-O), 766; ^1^H NMR (DMSO-d6, 400 MHz) $\delta$ (ppm): 3.55 (s, 6H, CH_3_O), 3.73 (s, 6H, CH_3_O), 5.06 (s, 2H, CH_benzyl_), 6.62–6.64 (d, J = 8 Hz, 2H, H_aromatic_), 6.87–6.89 (dd, J = 8 Hz, 2H, H_aromatic_), 6.94–6.98 (dd, J = 8, 2H, H_aromatic_), 7.07 (br.s, 4H, NH_2_), 7.11–7.13 (d, J = 8.4 Hz, 2H, H_aromatic_), 7.81–7.83 (d, J = 8.4 Hz, 2H, H_aromatic_) (see SI, Figs. S16–S18).

## Conclusion

In the current study, we introduced dendritic silica nanomaterials (KCC-1) as a mild, easy, efficient, high surface area (297 m^2^ g^−1^), high activity and stability catalyst for the one-pot synthesis of 2-amino chromenes by multicomponent reactions under ultrasonic irradiation. This enhancement activity was explained on the basis of high surface area and the excellent accessibility of the active sites due to the open and flexible fibrous structure of KCC-1 as well as present the high number of amino groups on the surface of the catalyst. Furthermore, ultrasonic wave radiations were found to have a beneficial effect on the reduction of activation energy for the synthesis of compounds, indicating their superiority over the thermal method with respect to the yields and reaction times. The catalyst showed excellent efficiency and could convert > 92% of the substrates for target molecules. We believe, this method offers several advantages including heterogeneous, easy separation, high surface area, reusability, resistance, and lower loading of the catalyst under ultrasonic irradiation. Also, high yield of products in low reaction times, simple experimental workup procedure, easy product separation, and purification are other advantages for this method. Investigation and fabrication of heterogeneous catalysts and their application in chemical reactions are an important field of chemical researches. Therefore, with the mentioned innovation, this catalyst can be considered as a new class of heterogeneous catalysts.

## Supplementary Information

Supplementary Information.
