# Supplementary material for: Silica nanospheres KCC-1 as a good catalyst for the preparation of 2-amino-4H-chromenes by ultrasonic irradiation
Source: Sci Rep. 2022 Feb 11;12:2381. doi: 10.1038/s41598-022-05993-3 (PMC8837639; doi:10.1038/s41598-022-05993-3)
Supplement: Supplementary file 1 — Supplementary Information. [file 41598_2022_5993_MOESM1_ESM.docx]

**Silica Nanospheres KCC-1 As A Good Catalyst for The Preparation of 2-Amino-4H-Chromenes by Ultrasonic Irradiation**

**Hourieh Sadat Oboudatian, Javad Safaei Ghomi***

*Department of Organic Chemistry, Faculty of Chemistry, University of Kashan, Kashan, 51167, I. R. Iran*

**Corresponding Author Tel.: +98 31 55912385; fax: +98 31 55552935; *E-mail: safaei@kashanu.ac.ir*

**Table of Content**

| **S.No** | **Content** | **Page Numbers** |
| --- | --- | --- |
| **1** | Characterization data | 2-8 |
| **2** | References | 9 |
| **3** | Spectral data of the new compounds | 10-18 |

**Characterization data**

**Physical and spectroscopic data of all products**

***2-(4-nitrobenzylidene)malononitrile (Ι)***

96%, yellow solid, ^1^H NMR (DMSO-*d_6_*, 400 MHz) $\delta$ (ppm): 8.11-8.13 (d, *J* = 8.8 Hz, 2H, H_aromatic_), 8.42-8.44 (d, *J* =8.8 Hz, 2H, H_aromatic_), 8.71 (s, 1H, H_vinyl_).

***3,9-diamino-1,7-bis(3-hydroxyphenyl)-1,7-dihydrochromeno[8,7-h]chromene-2,8-dicarbonitrile (1)***

90%, Yellow solid, m.p. >300 °C (decomp.), IR (KBr) ν (cm^-1^): 3444 (NH_2_), 3301 (NH_2_,OH), 2192(CN), 1652 (NH_2_ bending), 1596, 1455, 1386, 1280, 1246, 1187, 1081 (C-O), 886, 761; ^1^H NMR (DMSO-*d_6_*, 400 MHz) $\delta$ (ppm): 4.78 (s, 2H, CH_benzyl_), 6.67-6.69 (m, 4H, H_aromatic_), 7.07-7.10 (m, 4H, H_aromatic_), 7.13 (br.s, 4H, NH_2_), 7.22-7.24 (m, 2H, H_aromatic_), 7.86-7.88 (dd, J=8.4 Hz, 2H, H_aromatic_), 9.35 (s, 2H, OH). ^13^C NMR (DMSO-*d_6_*, 400 MHz) $\delta$ (ppm): 45.63, 55.15, 115.55, 116.42, 119.32, 120.62, 122.25, 126.54, 127.91, 129.17, 131.38, 142.97, 146.44, 154.46, 160.57. Anal. calcd for C_30_H_20_N_4_O_4_, % C, 71.99; H, 4.03; N, 11.19; Found, %: C, 70.86; H, 3.98; N, 10.85.

***3,9-diamino-1,7-bis(2-hydroxyphenyl)-1,7-dihydrochromeno[8,7-h]chromene-2,8-dicarbonitrile (2)***

85%, Yellow solid, m.p. >300 °C (decomp.), IR (KBr) ν (cm^-1^): 3467 (NH_2_), 3332 (NH_2_ ,OH), 3196, 2192(CN), 1650 (NH_2_ bending), 1595, 1461, 1383, 1280, 1187, 1080 (C-O), 889, 799; ^1^H NMR (DMSO-*d_6_*, 400 MHz) $\delta$ (ppm): 5.11 (s, 2H, CH_benzyl_), 6.91-6.93 (d, J=7.2 Hz, 2H, H_aromatic_), 6.98-7.01 (d, J=8.8 Hz, 2H, H_aromatic_), 7.27 (s, 4H, NH_2_), 7.39-7.43 (dd, J=8 Hz, 2H, H_aromatic_), 7.66-7.68 (d, J=8.4 Hz, 2H, H_aromatic_), 7.77-7.80 (d, J=8.8 Hz, 2H, H_aromatic_), 8.18-8.20 (d, J=8.4 Hz, 2H, H_aromatic_), 10.30 (s, 2H, OH). ^13^C NMR (DMSO-*d_6_*, 400 MHz) $\delta$ (ppm): 40.41, 56.23, 111.97, 118.19, 119.77, 120.63, 124.18, 124.63, 127.18, 138.25, 142.44, 147.75, 148.69, 153.24, 160.07. Anal. calcd for C_30_H_20_N_4_O_4_, % C, 71.99; H, 4.03; N, 11.19; Found, %: C, 71.96; H, 4.43; N, 11.54.

***3,9-diamino-1,7-bis(4-isopropylphenyl)-1,7-dihydrochromeno[8,7-h]chromene-2,8-dicarbonitrile (3)***

82%, Yellow solid, m.p = 315-320 °C (decomp.), IR (KBr) ν (cm^-1^): 3492 (NH_2_), 3379 (NH_2_), 2198(CN), 1651 (NH_2_ bending), 1596, 1455, 1386, 1272, 1232, 1187, 1086 (C-O), 755; ^1^H NMR (DMSO-*d_6_*, 400 MHz) $\delta$ (ppm): 1.148 (s, 6H, CH_3_), 1.161 (s, 6H, CH_3_), 2.82 (m, 2H, CH) 4.84 (s, 2H, CH_benzyl_), 7.12 (br.s, 4H, NH_2_), 7.13-7.27 (m, 10H, H_aromatic_), 7.85-7.88 (dd, *J* = 8.4 Hz, 2H, H_aromatic_). ^13^C NMR (DMSO-*d_6_*, 400 MHz) $\delta$ (ppm): 23.82, 33.00, 40.37, 56.28, 116.73, 119.13, 119.21, 120.45, 122.43, 126.63, 127.12, 127.38, 127.44, 142.93, 143.04, 146.96, 160.02. Anal. calcd for C_36_H_32_N_4_O_2_, % C, 78.24; H, 5.84; N, 10.14; Found, %: C, 77.86; H, 5.53; N, 10.44.

***3,9-diamino-1,7-bis(4-methylphenyl)-1,7-dihydrochromeno[8,7-h]chromene-2,8-dicarbonitrile (4)***

85%, Yellow solid, m.p =310-320 °C (decomp.), IR (KBr) ν (cm^-1^): 3454 (NH_2_), 3325 (NH_2_), 3202, 2922, 2196(CN), 1659 (NH_2_ bending), 1596, 1500, 1388, 1282, 1238, 1187, 1084 (C-O), 854, 763; ^1^H NMR (DMSO-*d_6_*, 400 MHz) $\delta$ (ppm): 2.07 (s, 6H, CH_3_), 5.40 (s, 2H, CH_benzyl_), 7.09-7.13 (m, 2H, H_aromatic_), 7.22-7.29 (m, 8H, H_aromatic_ and NH_2_), 7.37-7.40 (m, 2H, H_aromatic_), 7.61-7.63 (dd, J=8 Hz, 2H, H_aromatic_), 7.86-7.88 (dd, *J* = 8.8 Hz, 2H, H_aromatic_). ^13^C NMR (DMSO-*d_6_*, 400 MHz) $\delta$ (ppm): 39.17, 40.02, 55.28, 117.15, 118.08, 120.04, 122.08, 122.22, 122.66, 127.03, 130.53, 134.62, 143.08, 147.55, 147.95, 160.20. Anal. calcd for C_32_H_24_N_4_O_2_, % C, 77.40; H 4.87; N, 11.28. Found, %: C, 75.86; H, 4.03; N, 11.14.

***3,9-diamino-1,7-bis(4-nitrophenyl)-1,7-dihydrochromeno[8,7-h]chromene-2,8 dicarbonitrile (5)***

94%, Yellow solid, m.p. >300 °C (decomp.), IR (KBr) ν (cm^-1^): 3441 (NH_2_), 3336 (NH_2_), 3196, 2190(CN), 1655 (NH_2_ bending), 1597, 1527, 1387, 1350, 1281, 1187, 1082 (C-O), 800, 728; ^1^H NMR (DMSO-*d_6_*, 400 MHz) $\delta$ (ppm): 5.21 (s, 2H, CH_benzyl_), 7.28-7.30 (m, 4H, H_aromatic_), 7.34 (br.s, 4H, NH_2_), 7.60-7.73 (m, 6H, H_aromatic_), 7.90-7.92 (d, *J* =8 Hz, 2H, H_aromatic_), 8.12 (br.s, 2H, H_aromatic_). ^13^C NMR (DMSO-*d_6_*, 400 MHz) $\delta$ (ppm): 40.03, 55.26, 117.16, 118.11, 120.02, 122.05, 122.21, 122.67, 127.02, 130.53, 134.61, 143.09, 147.57, 147.95, 160.20. Anal. calcd for C_30_H_18_N_6_O_6_, % C, 64.52; H, 3.25; N, 15.05. Found, %: C, 63.45; H, 3.05; N, 15.45.

***3,9-diamino-1,7-bis(2,3-dimethoxyphenyl)-1,7-dihydrochromeno[8,7-h]chromene-2,8-dicarbonitrile (6)***

83%, Yellow solid, m.p >300 °C (decomp.), IR (KBr) ν (cm^-1^): 3430 (NH_2_), 3315 (NH_2_), 2195 (CN), 1654 (NH_2_ bending), 1599, 1477, 1386, 1284, 1077 (C-O), 766; ^1^H NMR (DMSO-*d_6_*, 400 MHz) $\delta$ (ppm): 3.55 (s, 6H, CH_3_O), 3.73 (s, 6H, CH_3_O), 5.06 (s, 2H, CH_benzyl_), 6.62-6.64 (d, *J=8* Hz, 2H, H_aromatic_), 6.87-6.89 (d, *J=8* Hz, 2H, H_aromatic_), 6.94-6.98 (dd, *J=8*, 2H, H_aromatic_), 7.07 (br.s, 4H, NH_2_), 7.11-7.13 (d, *J*=8.4 Hz, 2H, H_aromatic_), 7.81-7.83 (d, *J*=8.4 Hz, 2H, H_aromatic_). ^13^C NMR (DMSO-*d_6_*, 400 MHz) $\delta$ (ppm): 35.55, 55.53, 60.19, 60.27, 111.63, 116.55, 118.76, 120.52, 121.09, 122.36, 124.18, 126.70, 138.33, 143.05, 146.09, 146.21, 152.43, 160.16. Anal. calcd for C_34_H_28_N_4_O_6_, % C, 69.38; H, 4.79; N, 9.52. Found, %: C, 67.45; H, 3.95; N, 9.61.

***3,9-diamino-1,7-bis(3-chlorophenyl)-1,7-dihydrochromeno[8,7-h]chromene-2,8-dicarbonitrile (7):***

92%, Yellow solid, m.p = 310-315 °C (decomp.), IR (KBr) ν (cm^-1^): 3439 (NH_2_), 3327 (NH_2_), 3198, 2195(CN), 1649 (NH_2_ bending), 1595, 1474, 1385, 1280, 1187, 1080 (C-O), 893, 753; ^1^H NMR (DMSO-*d_6_*, 400 MHz) $\delta$ (ppm): 4.97 (s, 2H, CH_benzyl_), 7.17-7.35 (m, 10H, H_aromatic_), 7.24 (br.s, 4H, NH_2_), 7.88-7.90 (dd, *J* =8.4 Hz, 2H, H_aromatic_). ^13^C NMR (DMSO-*d_6_*, 400 MHz) $\delta$ (ppm): 37.77, 54.34, 117.06, 119.82, 122.56, 123.69, 126.42, 128.16, 129.27, 132.62, 133.07, 140.91, 141.00, 143.26, 160.14. Anal. calcd for C_30_H_18_Cl_2_N_4_O_2_, % C, 67.05; H, 3.38; N, 10.43; Found, %: C, 66.13; H, 2.97; N, 10.45.

***3,9-diamino-1,7-bis(4-hydroxyphenyl)-1,7-dihydrochromeno[8,7-h]chromene-2,8-dicarbonitrile (8)***

87%, Yellow solid, m.p. >300 °C (decomp.), IR (KBr) ν (cm^-1^): 3422 (NH_2_), 3323 (NH_2_,OH), 2923, 2180(CN), 1655 (NH_2_ bending), 1595, 1511, 1389, 1255, 1184, 1085 (C-O), 759; ^1^H NMR (DMSO-*d_6_*, 400 MHz) $\delta$ (ppm): 4.75 (s, 2H, CH_benzyl_), 6.67-6.69 (d, *J* = 8 Hz, 4H, H_aromatic_), 7.00-7.02 (d, *J* = 8 Hz, 4H, H_aromatic_), 7.06 (br.s, 4H, NH_2_), 7.17-7.20 (d, *J* = 8.8 Hz, 2H, H_aromatic_), 7.83-7.85 (d, *J* =8.4 Hz, 2H, H_aromatic_), 9.34 (s, 2H, OH). ^13^C NMR (DMSO-*d_6_*, 400 MHz) $\delta$ (ppm): 40.46, 55.04, 109.29, 111.23, 116.72, 118.52, 120.21, 124.06, 124.30, 127.45, 128.99, 142.85, 146.44, 153.29, 160.35. Anal. calcd for C_30_H_20_N_4_O_4_, % C, 71.99; H, 4.03; N, 11.19; Found, %: C, 71.26; H, 3.97; N, 11.37.

***3,9-diamino-1,7-diphenyl-1,7-dihydrochromeno[8,7-h]chromene-2,8-dicarbonitrile (9)***

89%, yellow solid, m.p. = 295-300 °C, IR (KBr) *ν* (cm^-1^): 3416 (NH_2_), 3284 (NH_2_), 3183, 3027, 2193(CN), 1648 (NH_2_ bending), 1597, 1496, 1453, 1386, 1281, 1186, 1080 (C-O), 740, 700; ^1^H NMR (DMSO-*d_6_*, 400 MHz) $\delta$ (ppm): 4.89 (s, 2H, CH), 7.15 (br.s, 4H, NH_2_), 7.20-7.33 (m, 12H, H_aromatic_) 7.86-7.89 (dd, *J* =8 Hz, 2H, H_aromatic_). ^13^C NMR (DMSO-*d_6_*, 400 MHz) $\delta$ (ppm): 40.31, 56.20, 116.79, 118.97, 119.04, 120.32, 122.47, 126.97, 127.07, 127.58, 127.62, 128.72, 142.94, 145.50, 159.99. Anal. calcd for C_30_H_20_N_4_O_2_, % C, 76.91; H, 4.30; N, 11.96; Found, %: C, 76.59; H, 4.28; N, 11.98.

***3,9-diamino-1,7-bis(4-chlorophenyl)-1,7-dihydrochromeno[8,7-h]chromene-2,8-dicarbonitrile (10)***

96%, yellow solid, m.p = 310-320 °C (decomp.), IR (KBr) *ν* (cm^-1^): 3463 (NH_2_), 3325 (NH_2_), 3193, 2196(CN), 1662 (NH_2_ bending), 1596, 1490, 1386, 1280, 1186, 1083 (C-O), 888, 799, 755; ^1^H NMR (DMSO-*d_6_*, 400 MHz) $\delta$ (ppm): 4.95 (s, 2H, CH), 7.20 (br.s, 4H, NH_2_), 7.23-7.27 (m, 6H, H_aromatic_), 7.35-7.39 (m, 4H, H_aromatic_), 7.86-7.88 (d, *J* = 8.8 Hz, 2H, H_aromatic_). ^13^C NMR (DMSO-*d_6_*, 400 MHz) $\delta$ (ppm): 38.37, 59.00, 115.36, 117.82, 120.66, 121.49, 124.92, 126.96, 127.17, 129.92, 132.07, 139.71, 143.64, 145.26, 160.34. Anal. calcd for C_30_H_18_Cl_2_N_4_O_2_, % C, 67.05; H, 3.38; N, 10.43; Found, %: C, 67.00; H, 3.34; N, 9.89.

***3,9-diamino-1,7-bis(2,4-dichlorophenyl)-1,7-dihydrochromeno[8,7-h]chromene-2,8-dicarbonitrile (11)***

94%, yellow solid, m.p. =320-325 °C (decomp.), IR (KBr) ν (cm^-1^): 3462 (NH_2_), 3325 (NH_2_), 3190, 2204(CN), 1670 (NH_2_ bending), 1596, 1489, 1390, 1283, 1180, 1083 (C-O), 867, 793, 758; ^1^H NMR (DMSO-*d_6_*, 400 MHz) $\delta$ (ppm): 5.40 (s, 2H, CH_benzyl_), 7.09-7.12 (d, *J* =8.8 Hz, 2H, H_aromatic_), 7.25-7.29 (m, 6H, H_aromatic_ and NH_2_), 7.37-7.41 (m, 2H, H_aromatic_), 7.63 (s, 2H, H_aromatic_), 7.86-7.88 (dd, *J* =10.8 Hz, 2H, H_aromatic_). ^13^C NMR (DMSO-*d_6_*, 400 MHz) $\delta$ (ppm): 35.57, 57.24, 116.06, 119.96, 121.76, 123.89, 125.92, 127.96, 129.67, 132.62, 133.47, 138.91, 141.00, 143.66, 159.84. Anal. calcd for C_30_H_16_Cl_4_N_4_O_2_, % C, 59.43; H, 2.66; N, 9.24; Found, %: C, 59.13; H, 2.57; N, 9.15.

***3,9-diamino-1,7-bis(4-bromophenyl)-1,7-dihydrochromeno[8,7-h]chromene-2,8-dicarbonitrile (12)***

93%, Yellow solid, m.p =300-310 °C (decomp.), IR (KBr) *ν* (cm^-1^): 3460 (NH_2_), 3325 (NH_2_), 3194, 2195(CN), 1660 (NH_2_ bending), 1595, 1486, 1386, 1281, 1186, 1078 (C-O), 888, 795; ^1^H NMR (DMSO-*d_6_*, 400 MHz) $\delta$ (ppm): 4.93 (s, 2H, CH_benzyl_), 7.17-7.21 (m, 6H, H_aromatic_), 7.22 (br.s, 4H, NH_2_), 7.49-7.52 (m, 4H, H_aromatic_), 7.86-7.88 (dd, *J* =8.4 Hz, 2H, H_aromatic_). ^13^C NMR (DMSO-*d_6_*, 400 MHz) $\delta$ (ppm): 40.10, 55.65, 116.90, 118.44, 118.50, 120.11, 120.18, 122.53, 127.02, 129.91, 129.94, 131.63, 142.95, 144.87, 159.95. Anal. calcd for C_30_H_18_Br_2_N_4_O_2_, % C, 57.53; H, 2.90; N, 8.95; Found, %: C, 57.13; H, 2.67; N, 9.00.

***3,9-diamino-1,7-bis(3-nitrophenyl)-1,7-dihydrochromeno[8,7-h]chromene-2,8-dicarbonitrile (13)***

87%, Yellow solid, m.p >300 °C (decomp.), IR (KBr) ν (cm^-1^): 3439 (NH_2_), 3327 (NH_2_), 3198, 2195(CN), 1649 (NH_2_ bending), 1595, 1474, 1385, 1280, 1187, 1080 (C-O), 893, 753; ^1^H NMR (DMSO-*d_6_*, 400 MHz) $\delta$ (ppm): 5.20 (s, 2H, CH_benzyl_), 7.32 (m, 8H, H_aromatic_ and NH_2_), 7.62-7.71 (m, 4H, H_aromatic_), 7.90-7.92 (m, 2H, H_aromatic_), 8.12 (br.s, 2H, H_aromatic_). ^13^C NMR (DMSO-*d_6_*, 400 MHz) $\delta$ (ppm): 39.43, 57.26, 116.71, 120.61, 124.62, 124.95, 125.21, 125.67, 127.52, 129.53, 137.61, 143.19, 146.57, 147.55, 160.10. Anal. calcd for C_30_H_18_N_6_O_6_, % C, 64.52; H, 3.25; N, 15.05. Found, %: C, 62.95; H, 2.85; N, 14.50.

***3,9-diamino-1,7-bis(4-methoxyphenyl)-1,7-dihydrochromeno[8,7-h]chromene-2,8-dicarbonitrile (14)***

87%, Yellow solid, m.p =300-305 °C (decomp.), IR (KBr) ν (cm^-1^): 3456 (NH_2_), 3330 (NH_2_), 3191, 2195(CN), 1659 (NH_2_ bending), 1596, 1388, 1282, 1187, 1084 (C-O), 817; ^1^H NMR (DMSO-d6, 400 MHz) $\delta$ (ppm): 3.70 (s, 6H, CH_3_O), 4.83 (s, 2H, CH_benzyl_), 6.85-6.87 (d, *J* = 8.4, 4H, H_aromatic_), 7.09 (br.s, 4H, NH_2_), 7.12-7.15 (d, *J* = 8.4, 4H, H_aromatic_), 7.18-7.20 (d, *J* =8 Hz, 2H, H_aromatic_), 7.84-7.86 (d, J=8.8 Hz, 2H, H_aromatic_). ^13^C NMR (DMSO-*d_6_*, 400 MHz) $\delta$ (ppm): 38.95, 55.73, 60.27, 112.53, 113.95, 117.66, 120.82, 121.89, 122.46, 126.78, 127.80, 129.33, 143.05, 146.09, 158.43, 160.26. Anal. calcd for C_32_H_24_N_4_O_4_, % C, 72.72; H, 4.58; N, 10.60. Found, %: C, 72.68; H, 4.51; N, 10.62.

***3,9-diamino-1,7-bis(3,4-dimethoxyphenyl)-1,7-dihydrochromeno[8,7-h]chromene-2,8-dicarbonitrile (15)***

85%, Yellow solid, m.p >300 °C (decomp.), IR (KBr) ν (cm^-1^): 3447 (NH_2_), 3385 (NH_2_), 3.202, 2189(CN), 1660 (NH_2_ bending), 1601, 1512, 1389, 1270, 1195, 1140, 1081 (C-O), 1020, 802, 766; ^1^H NMR (DMSO-*d_6_*, 400 MHz) $\delta$ (ppm): 3.68 (s, 6H, CH_3_O), 3.69 (s, 6H, CH_3_O), 4.80 (s, 2H, CH_benzyl_), 6.70-6.72 (d, J=8, 2H, H_aromatic_), 7.01-7.06 (m, 6H, H_aromatic_ and NH_2_), 7.38-7.40 (dd, 2H, H_aromatic_), 7.63-7.65 (d, *J* = 8 Hz, 2H, H_aromatic_), 7.74-7.77 (d, *J* = 8.4 Hz, 2H, H_aromatic_). ^13^C NMR (DMSO-*d_6_*, 400 MHz) $\delta$ (ppm): 37.55, 55.83, 60.19, 61.27, 116.63, 117.75, 119.66, 119.92, 120.96, 122.76, 125.18, 138.73, 143.55, 146.89, 147.21, 156.43, 160.16. Anal. calcd for C_34_H_28_N_4_O_6_, % C, 69.38; H, 4.79; N, 9.52. Found, %: C, 68.85; H, 4.15; N, 9.50.

**References**

- Shestopalov, A.M., Emelianova, Yu.M., Nesterov, V.N. One_step synthesis of substituted 2-amino-4H-chromenes and 2-amino-4H-benzo[f]chromenes. Molecular and crystal structure of 2-amino-3-cyano-6-hydroxy-4-phenyl-4H-benzo[f]chromene. *Russ. Chem. Bull.* **51**, 2238-2243. <https://doi.org/10.1023/A:1022135402451> (2002).
- Hosseini-sarvari, M., Shafiee-haghighi, S. Multi-component synthesis of 2-amino-4H-chromenes catalysed by nano ZnO in water, *Collect. Czech. Chem. Commun.* **76**, 1285–1298. https://doi.org/[10.1135/cccc2011050](http://dx.doi.org/10.1135/cccc2011050) (2011).
- Wang, X., Shi, D., Yu, H., Wang, G., Tu, S. Synthesis of 2-Aminochromene Derivatives Catalyzed by KF/Al_2_O_3_, *Synth. Commun.* **34**, 509–514. <https://doi.org/10.1081/SCC-120027291> (2004).
- El-Taweel, F.M.A., Elagamey, A.A., Elmougy, Sh.M. Studies on Substituted Heteroarenes: New Synthesis of Substituted Pyrrole, Pyridine, Pyrazolo[4,3-bpyridine, Pyrano [3,2-c]quinoline, Benzo [f] chromene, Benzo[h] chromene, Chromeno [8,7-h]chromene, Chromeno[6,5-f] chromene and 2H-chromene Derivatives. Egypt. J. Chem. **54**, 703-721 (2011).

**Spectral data of the new compounds**

***3,9-diamino-1,7-bis(3-hydroxyphenyl)-1,7-dihydrochromeno[8,7-h]chromene-2,8-dicarbonitrile (1)***


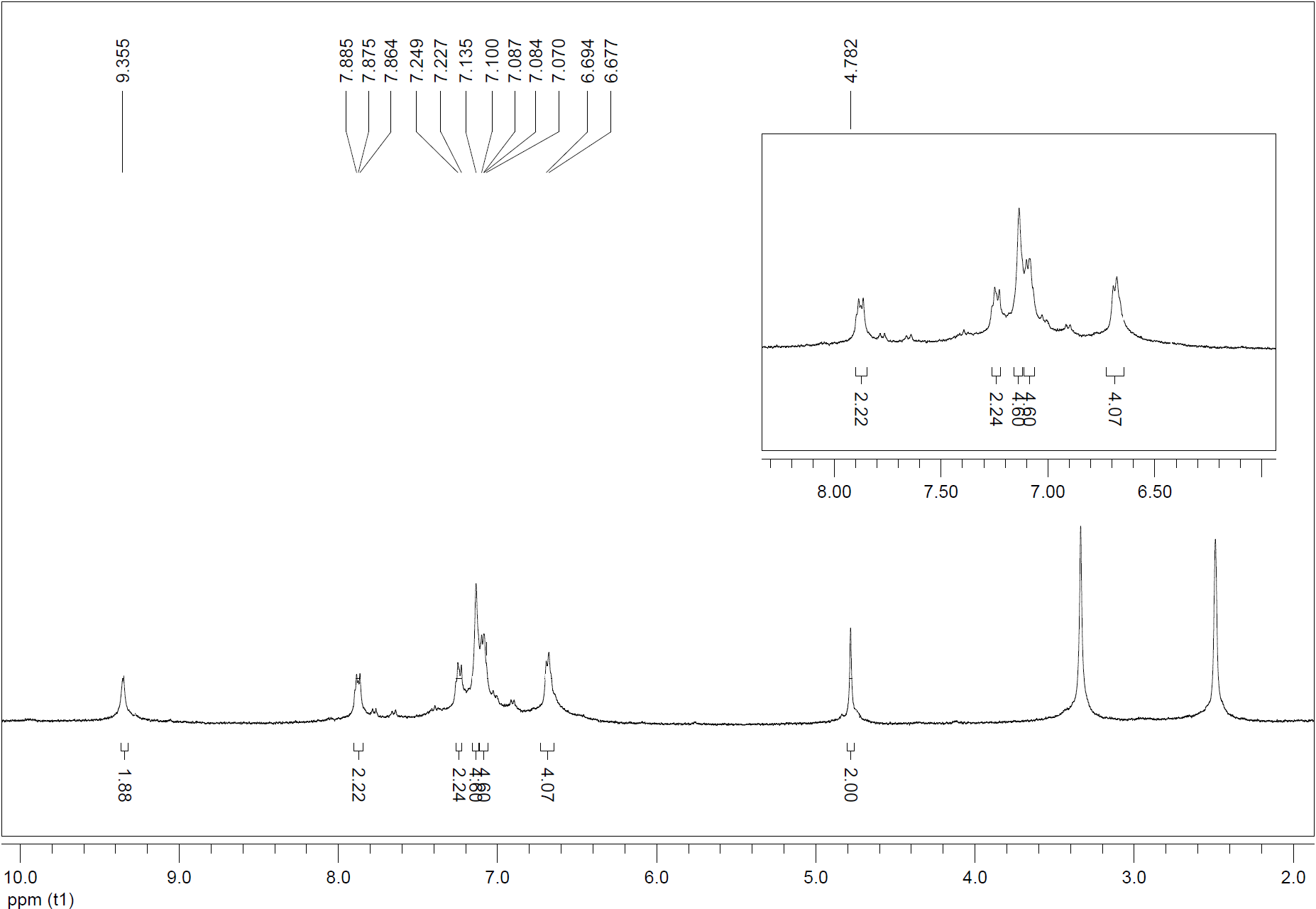

The ^1^H NMR spectrum of compound 4

Figure S1. ^1^H NMR spectrum of compound 1


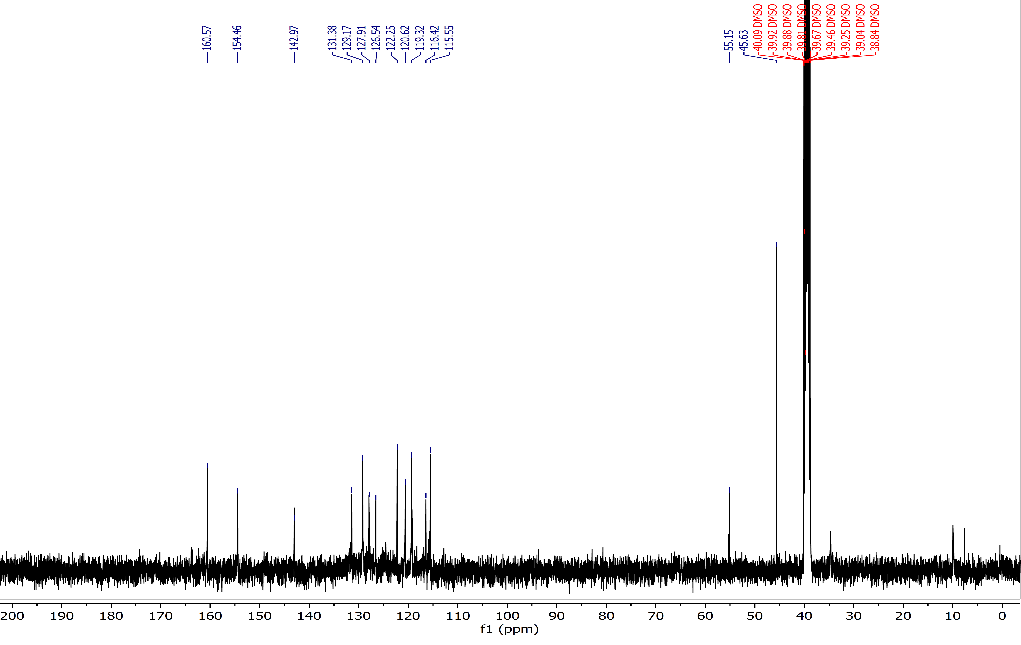


Figure S2. ^13^C NMR spectrum of compound 1


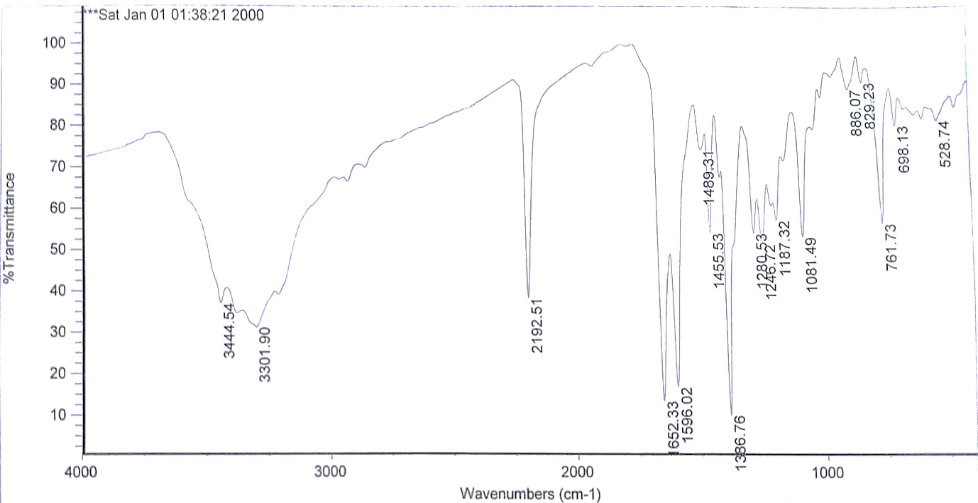


Figure S3. FT-IR spectrum of compound 1

***3,9-diamino-1,7-bis(2-hydroxyphenyl)-1,7-dihydrochromeno[8,7-h]chromene-2,8-dicarbonitrile (2)***

**
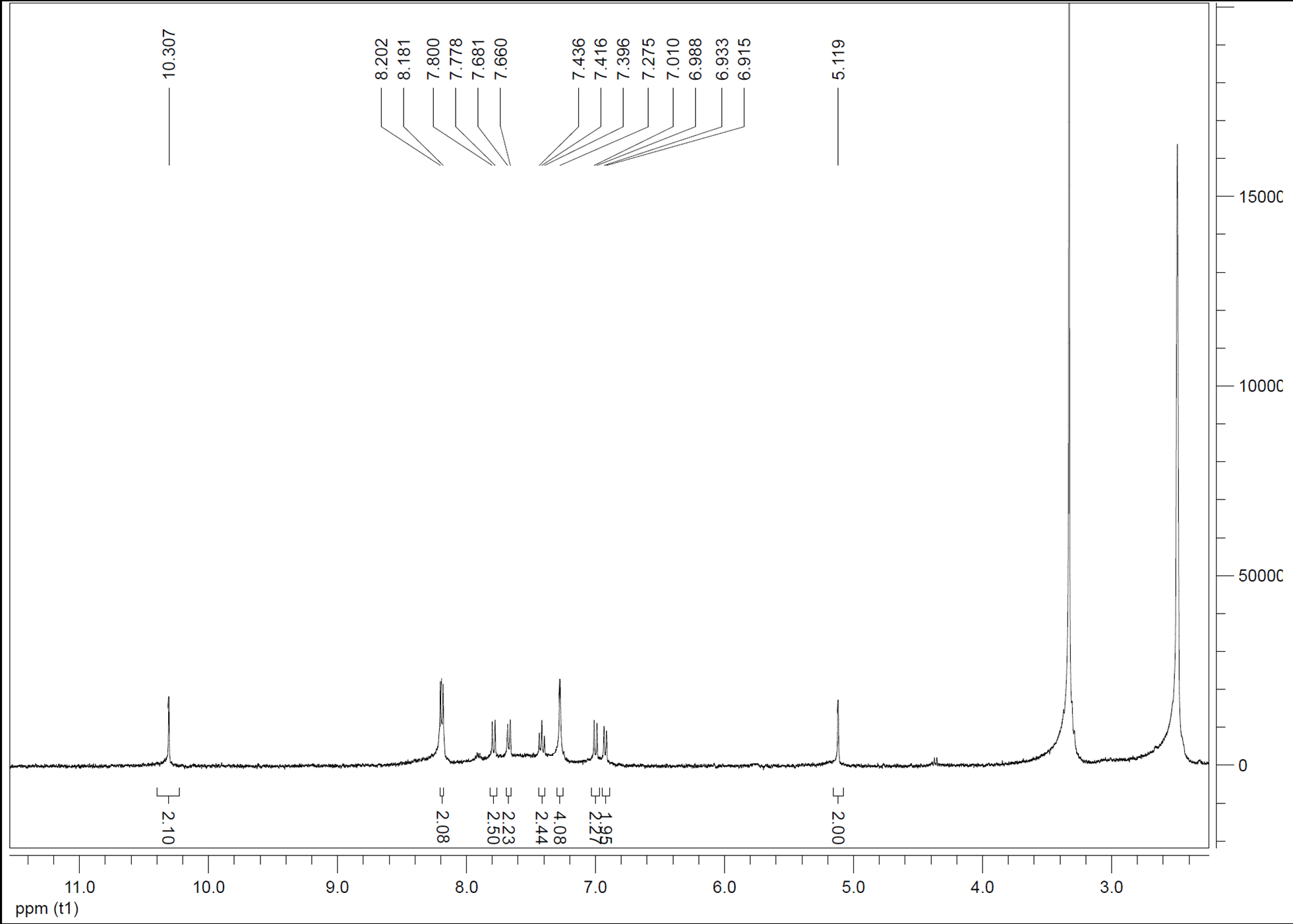
**

The ^1^H NMR spectrum of compound 4

Figure S4. ^1^H NMR spectrum of compound 2


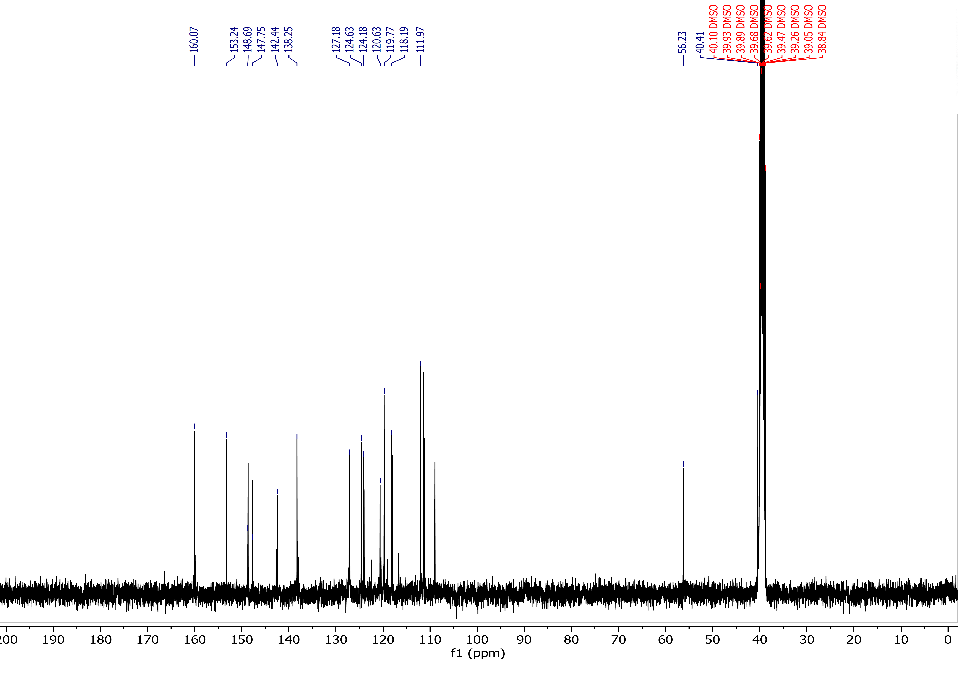


Figure S5. ^13^C NMR spectrum of compound 2


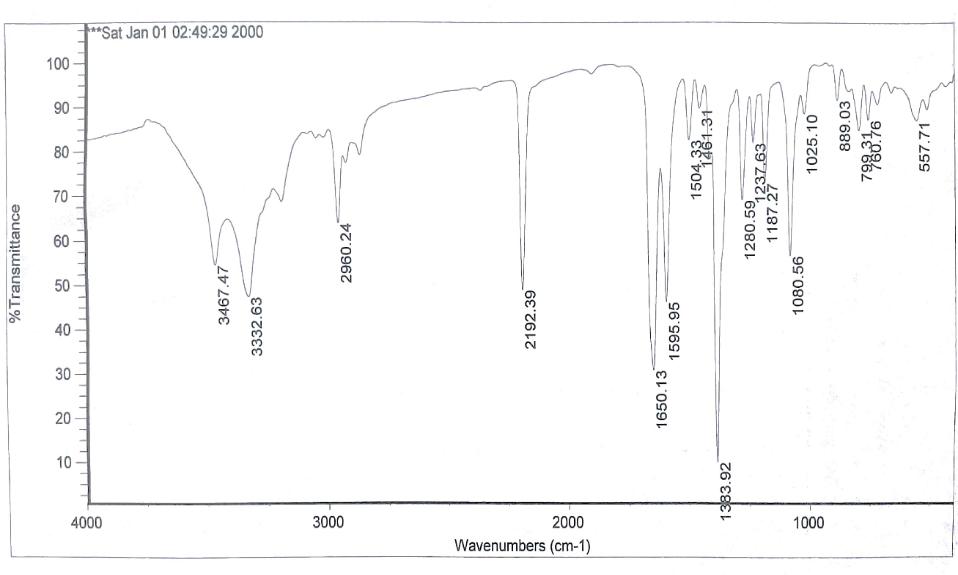


Figure S6. FT-IR spectrum of compound 2


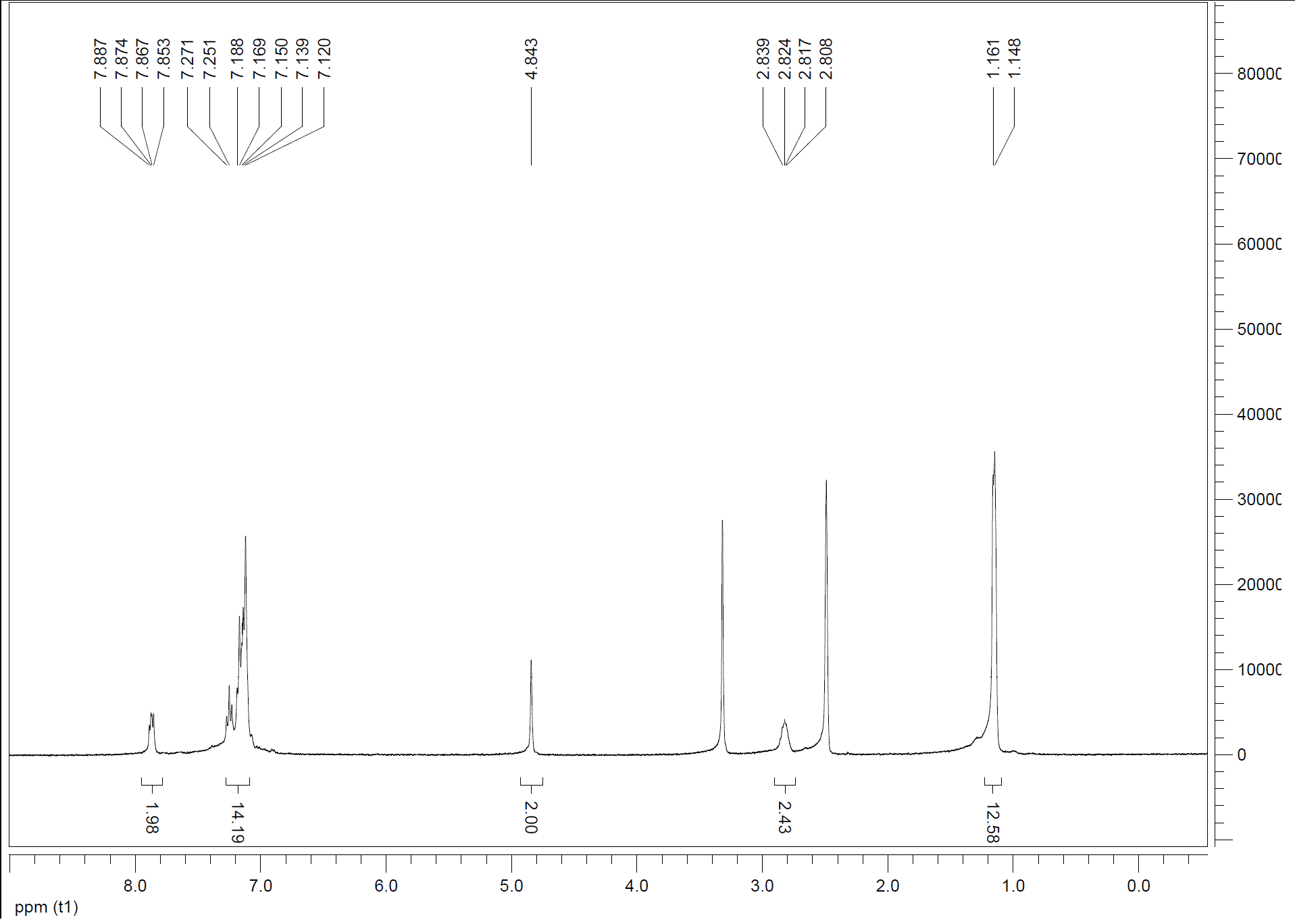
***3,9-diamino-1,7-bis(4-isopropylphenyl)-1,7-dihydrochromeno[8,7-h]chromene-2,8-dicarbonitrile (3)***

Figure S7. ^1^H NMR spectrum of compound 3


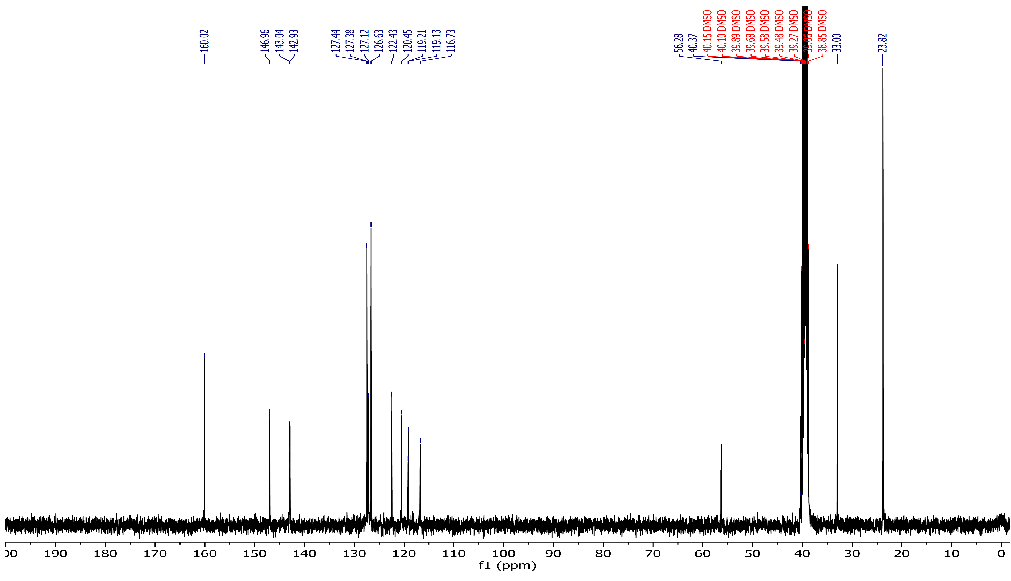


Figure S8. ^13^C NMR spectrum of compound 3


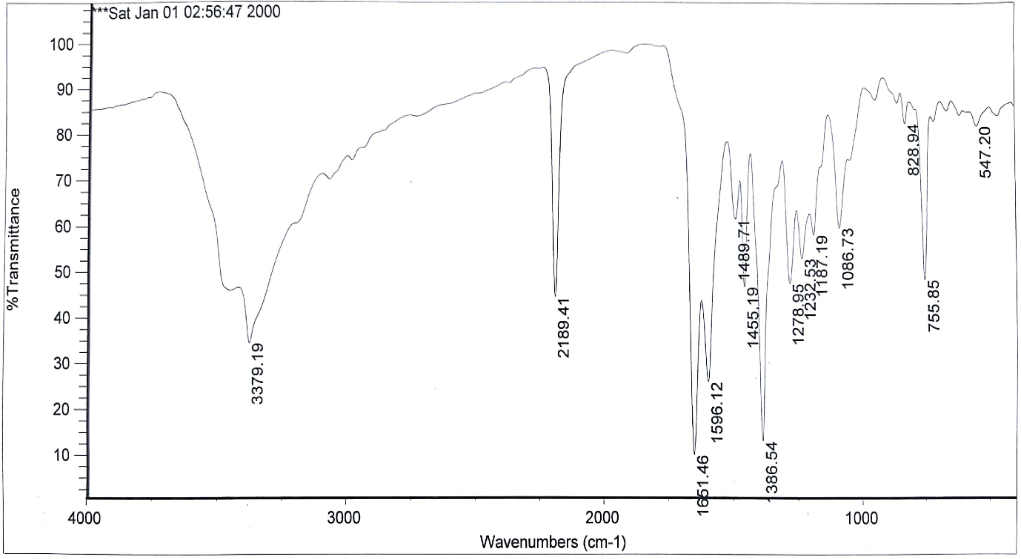


Figure S9. FT-IR spectrum of compound 3

***3,9-diamino-1,7-bis(4-methylphenyl)-1,7-dihydrochromeno[8,7-h]chromene-2,8-dicarbonitrile (4)***

**
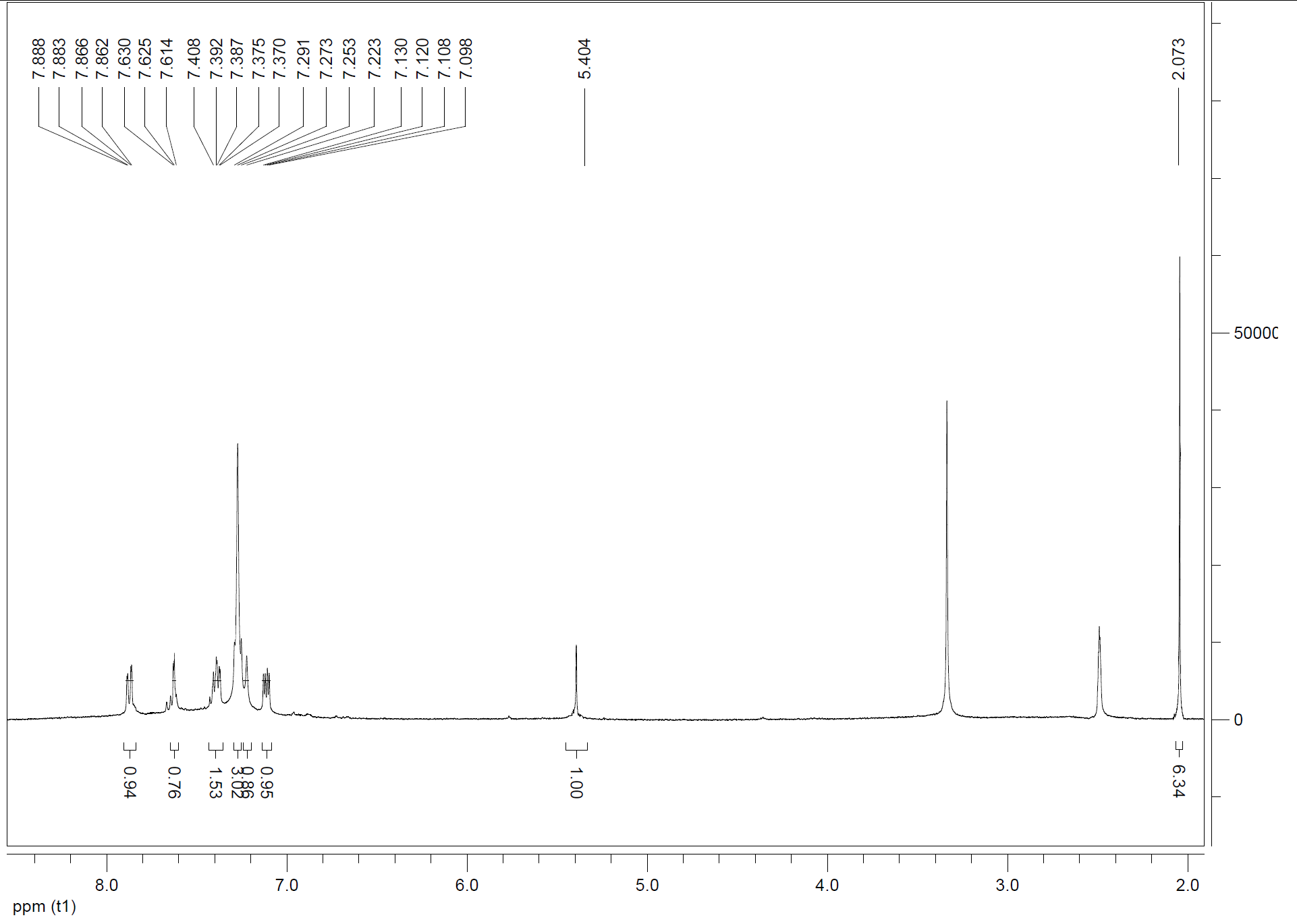
**

Figure S10. ^1^H NMR spectrum of compound 4


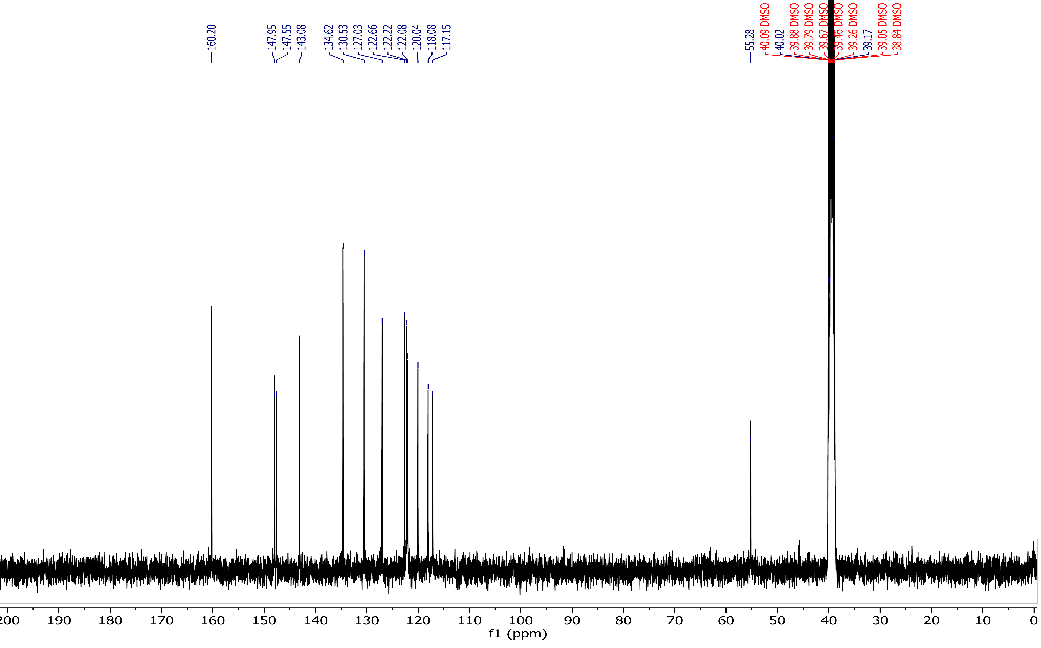


Figure S11. ^13^C NMR spectrum of compound 4


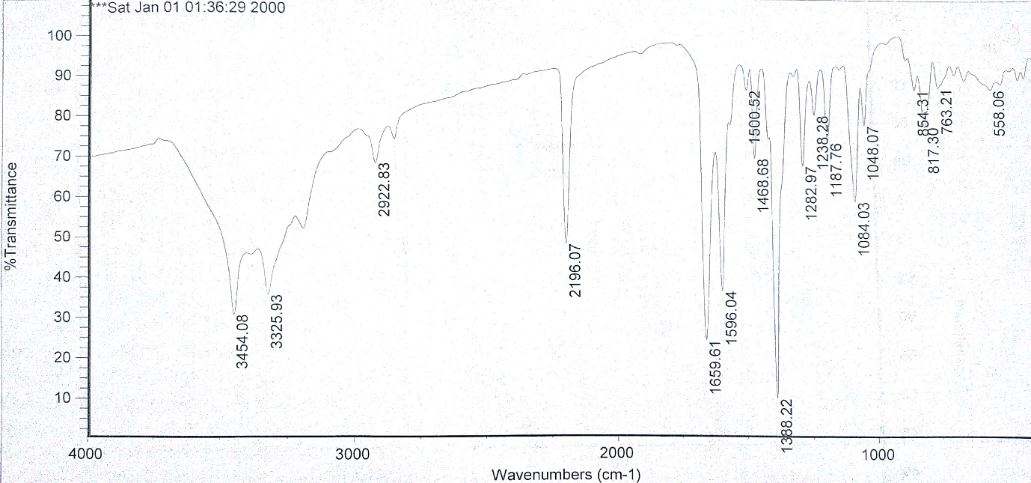


Figure S12. FT-IR spectrum of compound 4

***3,9-diamino-1,7-bis(4-nitrophenyl)-1,7-dihydrochromeno[8,7-h]chromene-2,8 dicarbonitrile (5)***


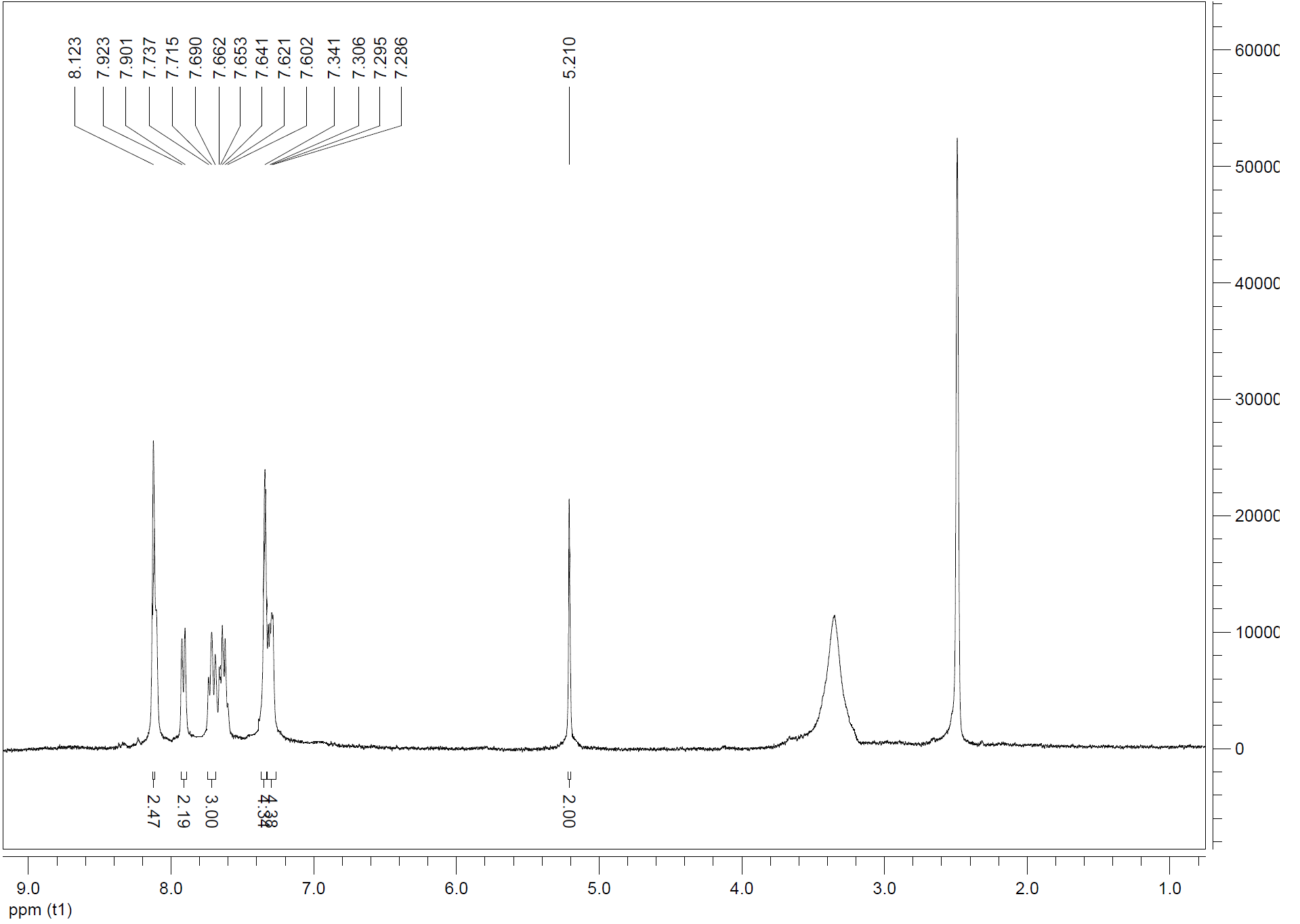

The ^1^H NMR spectrum of compound 4

Figure S13. ^1^H NMR spectrum of compound 5


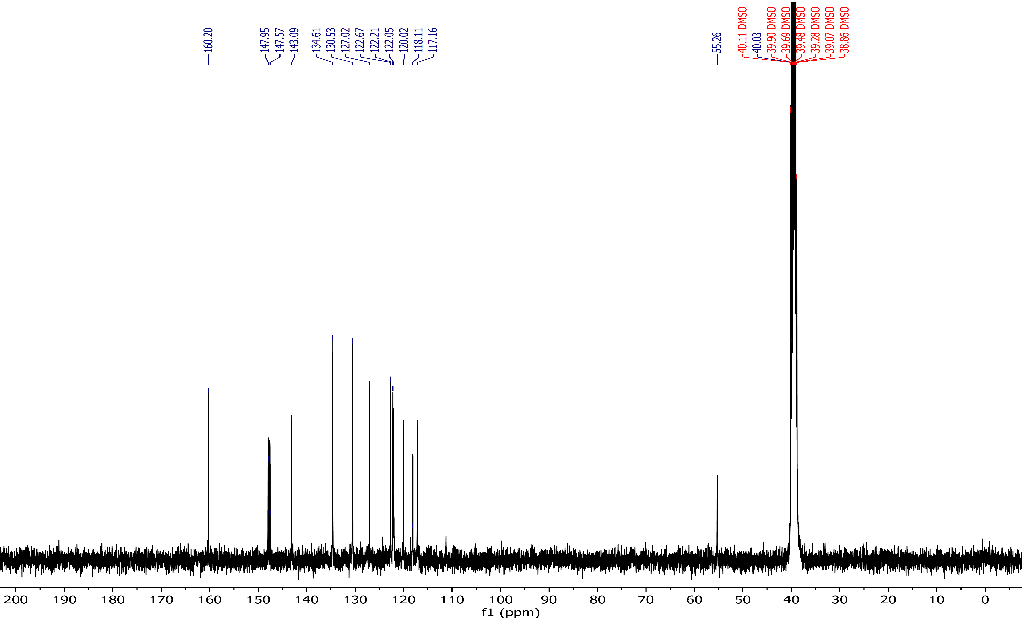


Figure S14. ^13^C NMR spectrum of compound 5


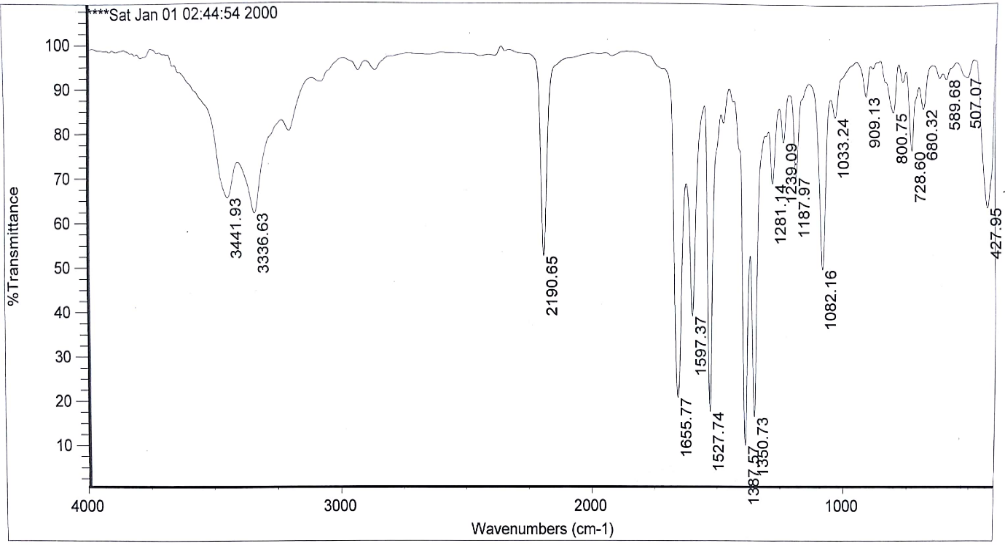


Figure S15. FT-IR spectrum of compound 5

***3,9-diamino-1,7-bis(2,3-dimethoxyphenyl)-1,7-dihydrochromeno[8,7-h]chromene-2,8-dicarbonitrile (6)***


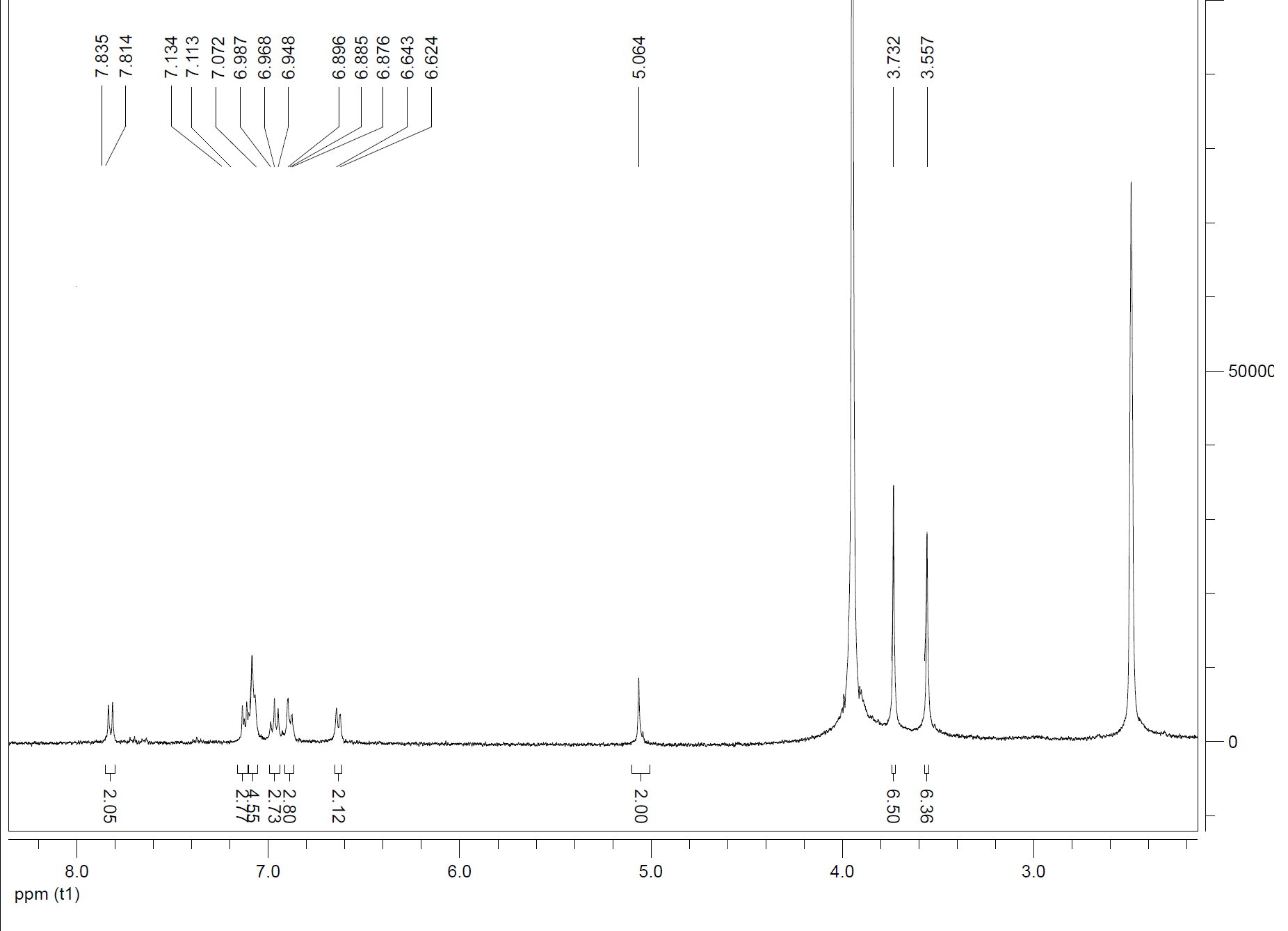

Figure S16. ^1^H NMR spectrum of compound 6


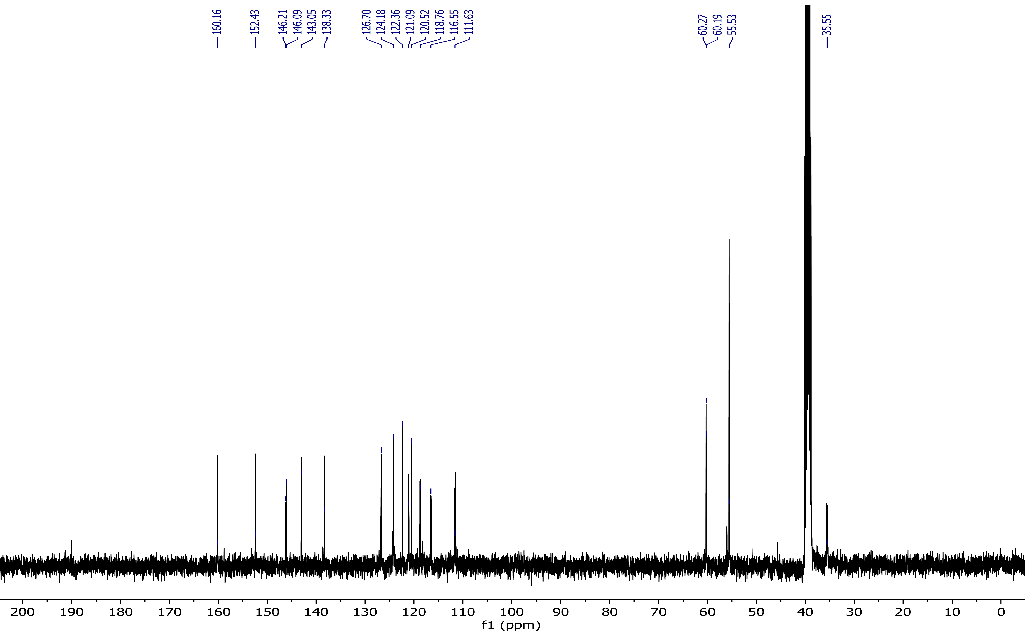


Figure S17. ^13^C NMR spectrum of compound 6


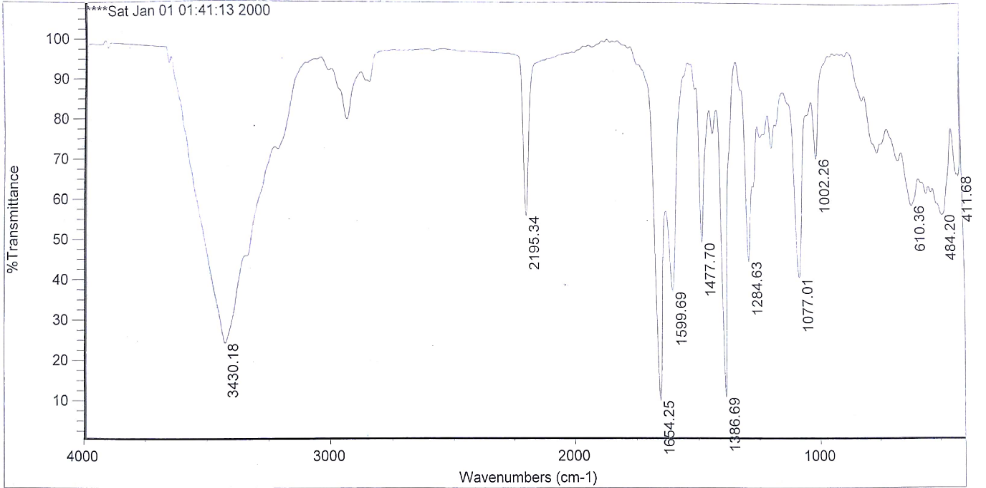


Figure S18. FT-IR spectrum of compound 6
